# Molecules in the Serotonin-Melatonin Synthesis Pathway Have Distinct Interactions with Lipid Membranes

**DOI:** 10.1021/acs.jpcb.4c08750

**Published:** 2025-02-28

**Authors:** Oskar Engberg, Debsankar Saha Roy, Pawel Krupa, Shankha Banerjee, Ankur Chaudhary, Albert A. Smith, Mai Suan Li, Sudipta Maiti, Daniel Huster

**Affiliations:** † Institute of Medical Physics and Biophysics, Medical Department, University of Leipzig, Härtelstr. 16/18, D-04107 Leipzig, Germany; ‡ Department of Chemical Sciences, Tata Institute of Fundamental Research, Homi Bhabha Road, Colaba, Mumbai 400 005, India; § Institute of Physics, Polish Academy of Sciences, Warsaw 02-668, Poland; ∥ Institute for Computational Science and Technology, Quang Trung Software City, Tan Chanh Hiep Ward, District 12, 729110 Ho Chi Minh City, Vietnam

## Abstract

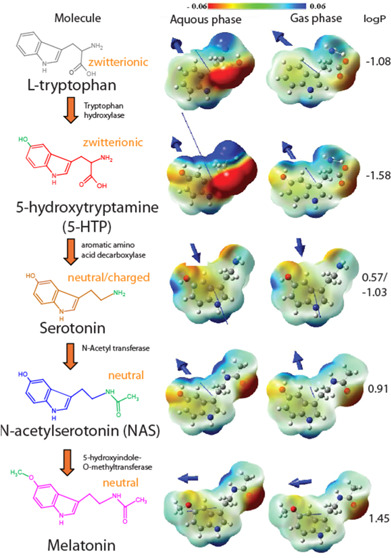

The neurotransmitter serotonin is involved in physiological processes such as appetite, sleep, and mood and diseases such as anxiety and depression. Traditionally, the effects of serotonin were thought to be initiated by binding to its target transmembrane receptors. It is also known that serotonin can bind directly to the membrane with high affinity and modulate lipid dynamics, lateral segregation of lipids, vesicular association, and membrane protein activity. We investigated if other small molecules in the serotonin metabolic pathway, some of which are known to be signaling molecules while some others are not, have similar membrane modulating effects. Therefore, we examined serotonin and several of its metabolites: 5-hydroxytryptophan (5-HTP), serotonin, *N*-acetylserotonin (NAS), and melatonin in model membranes mimicking synaptic membranes. Using ^2^H NMR spectroscopy of deuterated 1-palmitoyl-2-oleoyl-glycero-3-phosphocholine (POPC), we observed that all metabolites disorder the synaptic membrane-mimicking model membranes. The largest disordering effect was observed for NAS and the smallest for tryptophan. Using fluorescence correlation spectroscopy, it was found that only NAS promotes vesicular association similar to that of serotonin, while the others did not. Furthermore, we found that the serotonin metabolites differed in their membrane distribution by employing solid state ^1^H magic angle spinning nuclear Overhauser enhancement spectroscopy (NOESY) experiments in simple POPC membranes. Similar results were obtained in synaptic membrane mimics using molecular dynamics simulations. In conclusion, while the causal correlation between membrane modulation effects and membrane distribution for the serotonin metabolites remains elusive, this study suggests that small-molecule metabolites and drugs can have drastic biological effects mediated through the membrane. The finding that small changes in structure lead to very different membrane modulation and distributions suggests the possibility of developing membrane modulating drugs in the future.

## Introduction

Small molecular weight neurotransmitters such as serotonin are important for numerous physiological and pathophysiological processes in both the peripheral and central nervous systems.^1^ Serotonin regulates physiological processes such as appetite, mood, and sleep and also pathophysiological diseases such as depression, anxiety, schizophrenia, and obsessive compulsive disorder. However, the exact molecular mechanisms of action are not precisely known for all of these processes and conditions.^2^ Serotonin binds to a family of different membrane receptors including G protein-coupled receptors (GPCR).^3^ Several serotonergic drugs like selective serotonin reuptake inhibitors (SSRI) are on the market to treat diseases which are thought to be serotonin-related.^4^ They do not directly bind to serotonin receptors but block the serotonin reuptake in the synapse, leading to an increase in the extracellular synaptic concentration of serotonin.^4^ Subsequently, the higher bioavailability of serotonin leads to higher binding to serotonin receptors, but it is currently not clear if this is the only mechanism of action. Also, there are several unwanted side effects related to the prescription of SSRI.^5^ One mechanism to explain these side effects is related to the fact that lipophilic serotonergic drugs also partition into the lipid membrane and modulate the physical properties of the bilayer, which influences the function of other membrane proteins that are unrelated to direct serotonergic pathways.^6−11^ It is important to point out that under physiological conditions, the local concentration of serotonin can be very high and easily reach hundreds of millimolar.^12^ It is known that serotonin binds to membranes with high affinity.^13^ This leads to a fluidization of the membrane by decreasing lipid chain order concomitant with an increase in the area per lipid in both simple artificial membranes and synaptic model membranes.^14^ Atomic force microscopy (AFM) shows that serotonin also decreases the stiffness of the membrane.^14^ In addition, serotonin can modulate the domain size in membrane compositions prone to lateral segregation, such as in 1-palmitoyl-2-oleoyl-*sn*-glycero-3-phosphochocholine (POPC)/palmitoyl-sphingomyelin (PSM)/cholesterol 4:4:2 mixture, which, to some extent, mimics the composition of the outer plasma membrane leaflet.^6^ As the plasma membrane hosts a large number of membrane proteins involved in many biological processes, it is very likely that the membrane modulating effects of small lipophilic molecules could also affect the function of unrelated receptors. This was recently shown for the neuropeptide Y4 receptor, a GPCR that is activated by only three peptide ligands (NPY, PP and peptide YY), but not by small molecules. However, in the presence of moderate serotonin concentrations, a decrease in agonist binding affinity was observed.^8^ While synaptic vesicular exocytosis *in vivo* is a protein-dependent process, we found that in a synaptic membrane-mimicking model, serotonin could enhance association of lipid vesicles with a supported lipid bilayer.^15^ The enhanced vesicular association then led to more fusion events.

It has been shown that numerous lipophilic small molecules drugs can influence membrane acyl chain order,^16,17^ spontaneous curvature,^18^ membrane protein function,^8,19,20^ and membrane domain size.^7^ Besides serotonin and similar neurotransmitters, such effects have been shown for instance for kinase inhibitors,^21^ local anesthetics,^22^ receptor ligands,^23^ statins,^24^ and other small molecular weight drugs. Likely, almost all lipophilic molecules could influence membrane properties to some extent, which is medically relevant as most commercially used clinical drugs are small molecules.^25^ Although they have been developed to selectively target proteins, if high enough concentrations are reached, they will indirectly affect the membrane proteins through physical modulation of membrane properties, possibly having biologically relevant effects. Besides the related side effects, such membrane-mediated action could provide a mechanism of action for pharmacological interference by new drugs that directly target the membrane instead of a receptor.^26^ Therefore, it is important to understand which functional groups most significantly determine this membrane modulation. Serotonin, which has several membrane modulating properties, is only one of several serotonin metabolites. One of them, the hormone melatonin, is also an important neurotransmitter involved in circadian rhythm and acting as an antioxidant.^27^ Melatonin has also been shown to modulate membrane domain size similar to serotonin in complex membranes prone to lateral segregation.^28,29^ Furthermore, several serotonin metabolites compress lipid monolayers.^11^ Therefore, it is of interest if these and other serotonin-derived molecules also modulate membrane properties, and how their membrane modulation may differ from each other.

**Figure 1:**
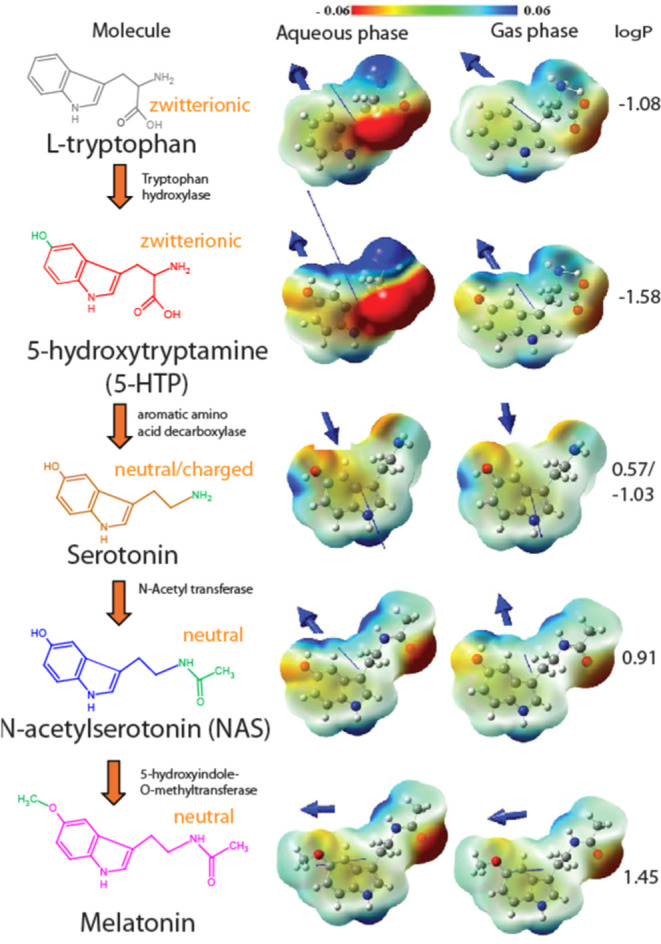
Schematic representation of the different serotonin metabolites. The charge state of the molecules at physiological pH is indicated in orange. Next to the arrows, the enzyme in the metabolic pathway is shown. The functional groups differing from the last metabolite are depicted in green. QM calculations showing charge distribution in color in the elementary charge unit where ±1 is of the elementary charge (*e* = ± 1.6 × 10^–19^ As). The direction of the dipole moment is indicated as a blue arrow where the length of the arrow is proportional to its strength. The arrowheads depicting the direction of the dipole moments have been zoomed in on the inset for visual guidance. Calculated log P values are shown at the right of each molecule.

We studied this by selecting serotonin and its metabolites *N*-acetylserotonin (NAS) and melatonin, which exhibit important biological effects. In addition, we included the precursors in the serotonin metabolic pathway tryptophan and 5-hydroxytryptophan (5-HTP). We use the term “serotonin metabolites” through the text to refer to all of these molecules. For the conversion of tryptophan, it is first hydroxylated in the aromatic ring by tryptophan hydroxylase to 5-HTP. 5-HTP is further metabolized to serotonin by decarboxylation by the enzyme aromatic amino acid decarboxylase. Serotonin is modified to NAS by *N*-acetyltransferase. Melatonin is formed by methylation of the 5-OH group by the enzyme 5-hydroxyindole-O-methyltransferase (Figure 1). This natural sequence of synthesis gives us the opportunity to characterize which functional group on the basic chemical structure leads to the strongest effects on the membrane. Small alterations in chemical structure are known to be very important for modulating membrane properties.^30−32^ The hypothesis is that individual modulations on the indole ring could fine-tune membrane binding, distribution, and localization of a given small molecule and individually influence metabolic pathways indirectly. This information could be used to plan and synthesize membrane modulating small molecules, e.g., serotonin derivatives that do not activate the serotonin receptor but modulate the membrane. To study possible membrane modulation by the serotonin metabolites, we employed several methods to study this in POPC and membranes mimicking the synaptic lipid distribution (POPC/1-palmitoyl-2-oleoyl-*sn*-glycero-3-phosphoethanolamine (POPE)/1-palmitoyl-2-oleoyl-*sn*-glycero-3-phospho-l-serine (POPS)/cholesterol in a molar ratio 3/5/2/5). When experimentally possible, small unilamellar vesicles (SUVs), mimicking synaptic vesicles in size, were used; otherwise supported bilayers (SLB) for AFM and multilamellar vesicles (MLVs) for NMR experiments were used. We found that the serotonin metabolites differ in their membrane distribution and modulation, and only some seem to lead to biologically interesting effects such as increased vesicular association.

## Material and Methods

### Materials

The lipids POPC, POPC-*d*_31_, POPE, and POPS were purchased from Avanti Polar Lipids Inc. (Alabaster, USA). Cholesterol, tryptophan, 5-HTP, NAS, serotonin-hydrochloride, and melatonin were purchased from Merck (Darmstadt, Germany). Chloroform and methanol were of high-performance liquid chromatography grade. All of the chemicals were used without any additional purification. Supported bilayers were formed on mica (grade V4 muscovite) as support purchased from SPI supplies (West Chester). Cover glasses with no 1.5 were acquired from Corning. All other chemicals were purchased from Merck (Darmstadt, Germany). The Milli-Q water (conductivity 18.2 MΩ cm^–1^) was obtained from a Milli-Q gradient system (Millipore, Germany).

### Quantum Chemical Calculations

*Ab initio* quantum chemical calculations were carried out to investigate the distribution of the electron cloud over the molecules in the aqueous and gaseous media, separately. We used Gaussian16 for geometry optimization and single-point energy calculations using B3LYP theory and 6-311++G (d,p) basis set. Electrostatic potential charges on the optimized geometry were obtained using the ChelpG module and subsequently used to generate the electron density map on each molecule in both aqueous medium and gaseous medium.^33,34^

### Preparation of SUVs

The lipid powders were dissolved in chloroform and mixed. For mimicking of synaptic membranes, a POPC/POPE/POPS/cholesterol mixture in a 3:5:2:5 molar ratio was prepared. The solvent was evaporated by rotating the vial under an Iolar argon gas to create a thin lipid film. This film was placed in a vacuum desiccator for ∼24 h to remove all organic solvents, ensuring it was dry. The dried film was then rehydrated with HEPES buffer (20 mM HEPES, pH 7.4, and 150 mM NaCl) to produce a lipid suspension. The solution was vortexed for ∼20 min to form MLVs and then sonicated for 10 min to yield a clear solution of SUVs.

### Lipid Binding Experiment

We quantified the equilibrium fraction of molecules bound to SUVs using fluorescence lifetime data. Fluorescence lifetime of the probe in buffer and the fluorescence lifetime of samples containing 1 μM molecule and lipid vesicles (concentration of lipid ∼10 mg/mL) were measured. The fluorescence lifetime experiments were performed using a home-built time-correlated single photon counting (TCSPC) setup. A 295 nm ps 10 MHz pulsed laser was used for exciting the samples. The emission was collected in magic angle polarization in the wavelength range of 330–360 nm using a slit. The fluorescence lifetime of all probes depends on their environment. Their fluorescence decay in buffer is single exponential in nature, denoting only one fluorescence lifetime component (except tryptophan, which has 2 lifetime components in buffer). In the presence of lipid vesicles, two additional fluorescence lifetimes emerge (one shorter lifetime component at ∼650–1000 ps and another at ∼14–21 ns). The fraction bound to lipid vesicles can be obtained from the relative amplitudes of the new lifetimes in lipid vesicles. Tryptophan has two lifetimes in buffer (∼3 and ∼650 ps). In the presence of lipid vesicles, the relative amplitude of the shorter lifetime component increases, and one more lifetime component emerges around ∼20 ns. The fraction of tryptophan bound to lipid vesicles can be obtained by measuring the relative amplitude of the ∼20 ns lifetime component and the increase in the relative amplitude of the ∼650 ps lifetime component. We note that the extent of lipid binding-induced increase in the relative amplitude of the ∼650 ps component would be attenuated due to depletion of molecules present in the aqueous phase. The true lipid binding-induced increase in the relative amplitude of the shorter lifetime component can be obtained from additionally knowing the depletion of the fraction of molecules having lifetime ∼3 ns. All measurements were performed at 25 °C.

### Preparation of Supported Bilayers

The mica-supported lipid bilayers (SLB) and SUVs were prepared by using the vesicle fusion technique described in our previous article.^35^ SLBs were formed by adding 20 μL of 100 mM CaCl_2_ solution, 25 μL of SUVs, and 105 μL of HEPES buffer (20 mM HEPES, pH 7.4, 150 mM NaCl) together (all were preheated to 65 °C in a water bath) on a freshly cleaved mica glued to a glass Petri dish. This dish was kept in a water bath at 70 °C for 1 h. The vesicles fuse to form a SLB in this condition. The excess of unfused vesicles was removed by thoroughly rinsing the bilayer with HEPES buffer (20 mM HEPES, pH 7.4, 150 mM NaCl). The formation of SLB was confirmed by the AFM force indentation study.

### AFM Force Indentation

All AFM measurements were recorded using a commercial NanoWizard II system (JPK Instruments, Berlin, Germany). The AFM apparatus was fixed atop an Axiovert inverted microscope manufactured in Zeiss, Germany. Prior to every force experiment, the thermal noise approach was used to calibrate the sensitivity, determine the spring constant, and determine resonance frequencies (in both air and water).^36^ The cantilever with a spring constant of 0.03 N/m and a resonance frequency of 10–20 kHz was utilized in all of the force measurements. Following each experiment, measurements of the spring constant and the sensitivity were also made. Before and after the experiments, the sensitivity values were comparable within error. All of the AFM force experiments were performed on the supported bilayers which were formed on mica glued to a glass coverslip in a liquid cell. Up until the conclusion of the experiment, the bilayer stayed hydrated. The overall piezo-opezoidal displacement for all bilayer force trials was 1.0 μm. The piezo velocity was maintained at 0.5 μm·s^–1^ during both approach and retraction. Measurements of indentation were made in accordance with^37^. The force value, also referred to as the indentation force or breakthrough force (*F*_X_), is a measurement of the membrane’s stiffness. Each force experiment was conducted at various locations on the bilayer. Typically, 400–600 force curves were recorded for each set. The force indentation curves were processed and analyzed by using JPK data processing software. The breakthrough force values were extracted from each approach curve to build the histogram for the measurements conducted at 25 °C.

### Fluorescence Correlation Spectroscopy for Intervesicle Association Measurement

We used fluorescence correlation spectroscopy (FCS) to assess intervesicular association, following our previously published protocol.^15^ Vesicle association can be estimated from the time scale of decay of the FCS traces of Nile red-labeled vesicles. Ten nM Nile red dye was incubated with a vesicle solution (∼10 nM vesicle concentration) for 20 min. The solution was then placed on a glass coverslip, and FCS measurements were performed. Each measurement was repeated at least 4 times, and the results are presented as the average ± SEM of these replicates. The measurements were performed by using a home-built FCS setup. In brief, a 488 nm laser beam was expanded and collimated through a 1:4 telescope setup. This collimated beam was focused into the sample with an apochromatic 60× water immersion objective (numerical aperture 1.2, Olympus, Center Valley, PA). Fluorescence was collected using the same objective and separated from the excitation beam with a 500 nm long-pass dichroic mirror (Chroma Technology, Rockingham, VT). The emission beam was focused onto a 15-μm core-diameter multimode optical fiber, filtered through a 607/70 nm bandpass emission filter (Chroma Technology, Rockingham, VT). The fiber acted as a confocal pinhole to eliminate out-of-focus fluorescence. The fluorescence was detected by a single photon avalanche photodiode (PerkinElmer, Waltham, MA) connected to the other end of the fiber. Data were collected and processed by using a hardware correlator (PicoHarp 300; PicoQuant, Berlin, Germany). The FCS data were fitted using a two-component, three-dimensional diffusion model with a triplet component (eq 1) in Origin 6.0 software.

Here, *G*(τ) is the autocorrelation function at lag time τ, *f* is the triplet component fraction, and τ_t_ is the triplet lifetime. τ_D1_ and τ_D2_ are the diffusion times for the two diffusing species in solution (free Nile red and Nile red bound to vesicles), *g*1 and *g*2 are their amplitudes, a is the structure parameter for the optical probe volume (assumed to be a Gaussian ellipsoid), and bl denotes the background signal. The parameters *z*_0_ and *w*_0_ are the focal volume’s (assumed to be a 3D Gaussian) characteristic length and width, respectively. Free rhodamine B dye served as a standard to calibrate the instrument. We obtained a diffusion time (τ_D_) of 27 μs for free rhodamine B in Hepes buffer (pH 7.4). The *R*_H_ for free rhodamine B was considered to be 0.58 nm.^38^ The diffusion times of the Nile red-labeled vesicles (τ_D2_) were converted to *R*_H_ by comparing their diffusion times with that of free rhodamine B in solution according to the following equations. Measurements were carried out at 25 °C.
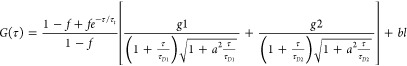
1where , , and  (Diffusion constant). Therefore,
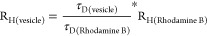


### Sample Preparation for Solid State NMR

Lipids were dissolved in MeOH/chloroform 1:1 (v/v) mixture, and serotonin metabolites (10 mol % for ^2^H NMR and 20 mol % for NOESY samples) were dissolved in MeOH and mixed with the lipid solutions. For mimicking synaptic membranes, a POPC/POPE/POPS/cholesterol mixture in a 3:5:2:5 molar ratio was prepared. A rotary evaporator at 40 °C was used to remove the solvent. Afterward, the samples, except tryptophan, were dissolved in cyclohexane and lyophilized overnight at vacuum to form a fluffy powder easy to hydrate. The tryptophan sample was dissolved in H_2_O and lyophilized for 72 h because of poor solubility in cyclohexane. All samples were hydrated to 50 wt % using a K_2_HPO_4_ buffer (20 mM K_2_HPO_4_, 100 mM NaCl, 0.1 mM EGTA pH 7.4) prepared with H_2_O (for ^2^H NMR samples) or D_2_O (for NOESY samples). Subsequently, the samples were freeze-thawed 10 times in liquid nitrogen or a water bath at 40 °C to form MLVs. Finally, the samples were transferred to inserts of 4 mm NMR rotors.

### Solid State NMR Measurements

NOESY experiments were performed on a Bruker Avance III 600 MHz NMR spectrometer (Bruker Biospin GmbH, Rheinstetten, Germany) using a high resolution 4 mm magic angle spinning (MAS) probe at 25 °C. A MAS frequency of 6 kHz with a π/2 pulse length of 4 μs was used. A ^2^H lock was used for the field stability. The terminal methyl peak at 0.885 ppm was used to calibrate the ^1^H NMR spectra. NOESY spectra with mixing times of 0.1, 100, 200, 300, and 500 ms were acquired. The spectra were assigned, and the volumes of the diagonal and cross peaks were integrated in Bruker Topspin 4.0. The cross-relaxation rates were calculated using the spin pair model using an in-house python script. NOESY histograms were plotted by calculating the average z distance of the different functional groups of POPC from a molecular dynamics (MD) simulation.^39^ The *z*-axis was referenced to 0 as the middle point of the terminal CH_3_ group distribution.

^2^H NMR spectra were acquired at a temperature of 25 °C on a Bruker Avance I 750 MHz spectrometer equipped with a 4 mm magic angle triple channel gradient probe at a resonance frequency of 115.1 MHz. A quadrupolar echo sequence pulse program with two π/2 pulses of 2.5–4 μs length separated by a 30 μs delay was used for signal acquisition.^40^ A recycle delay of 1 s was used, and the spectral width was ±250 kHz. dePaking the spectra and calculating average order parameters and smoothed order parameters profiles were performed in Mathcad as described previously.^41,42^

### Molecular Dynamics Simulation Setup

MD simulations were conducted using Amber software, employing the ff19SB force field for tryptophan,^43^ GAFF2 for small molecules,^44^ the four-point OPC water model^45^ with co-optimized ions (0.15 M NaCl including counterions to neutralize the charge), and the Lipid21 force field for lipids.^46^ Six systems were studied: a reference lipid bilayer with water and ions and bilayers with serotonin, NAS, tryptophan, 5-HTP, and melatonin. A symmetrical bilayer was simulated, which contained 240 lipids (80 POPE, 48 POPC, 32 POPS, 80 cholesterol), 33 Cl^–^ ions, 64 Na^+^ ions, and approximately 12,000 water molecules, with the addition of either 2 or 20 small molecules. The system size after equilibration was approximately 7.9 × 7.9 × 9.8 nm^3^. The simulation was performed at 37 °C.

### Parameterization

Partial charges were derived using the RESP method, implemented in the sixth edition of the RED server development,^47^ based on geometry optimization in GAMESS^48^ with the Hartree–Fock 6-31G* basis function. Other parameters were adapted from the Amber ff19SB and GAFF2 force fields based on structural similarities to other well-tested systems. Lipid bilayers were generated using the CHARMM-GUI server^49,50^ and manually edited to include the specified number of neurotransmitter molecules. Topology and coordinate files were constructed using tLeap, part of AmberTools 24.^51^

### Equilibration and Production Runs

Each system underwent energy minimization with 5000 steps (1000 steepest descent and 4000 conjugate gradients) and was heated from 10 to 310 K over a 5 ps MD run. Proper density was achieved during a subsequent 0.1 ns MD simulation in the NPT ensemble. Temperature was controlled using Langevin dynamics, and pressure was maintained using the Berendsen barostat.^52^ Initial equilibration involved running systems without neurotransmitters for 1 μs in the NPT ensemble followed by a 1.5 μs production run. Equilibrated lipid bilayers were then used to add two noninteracting neurotransmitter molecules, equilibrated for 0.5 μs, and subjected to a 1.5 μs production run. This process was repeated with the addition of 18 more neurotransmitter molecules (20 in total) to the equilibrated systems from the previous step, providing a total simulation time of 6.5 μs for each lipid-bilayer–neurotransmitter systems.

### Analysis

Since the neurotransmitters were added to the solvent at the beginning of each simulation, only the equilibrated parts of the trajectories were analyzed (last 1.5 μs), where they reached their optimal positions. Initial analysis, such as reimaging and PDB trajectory generation, measurement of the periodic boundary box size, and determination of hydrogen bonds based on distance and angle criteria was performed using cpptraj, part of AmberTools24. In-house scripts were then used to measure the structural properties of the studied systems, such as distance and angle measurements of the molecules relative to the lipid bilayer center.

## Results

The structures of the different serotonin metabolites are shown in Figure 1 together with several structural parameters of the respective molecules.

We performed quantum mechanical calculations for all molecules to compare their physical properties in both the aqueous and gas phases (Figure 1). Since the gas phase approximates the hydrophobic lipid environment, changes in dipole moment between aqueous and gas phases may indicate how molecular properties change upon membrane insertion. However, in practice, the change in dipole moment would be much smaller because these molecules preferentially insert in the lipid headgroup/glycerol region, where the dielectric constant remains relatively high. The exact dipole moments and changes between the aqueous and the gas phases can be found in Table S1. The calculations revealed that tryptophan and 5-HTP had the highest dipole moments directed close to the normal of the indole ring. Serotonin, NAS, and melatonin show weaker dipole moments, which were almost coplanar with the plane of the indole ring. The high dipole moment of tryptophan and 5-HTP could make them orient along the dipole of the lipid headgroup possibly affecting membrane modulation.

We also calculated the octanol–water partition coefficient (logP) for all molecules, which represents a measure of their polarity and lipophilicity. The estimated logP values varied between −1.6 and +1.5 for the serotonin metabolites, which suggests that the molecules are quite hydrophilic (Figure 1). Nevertheless, serotonin binds to the membrane with high affinity as previously shown experimentally.^13,14^ From its logP value, it is not totally clear why serotonin has a high affinity for the membrane. Serotonin could form H-bonds in the membrane, but H-bonds found in the aqueous phase could be lost. In addition, potential disordering of the lipid chains would also unfavor enthalpic effects, so entropic contributions are the most likely explanation. To confirm the high membrane affinity for the other serotonin metabolites, we carried out lipid binding assays. All serotonin metabolites are weakly fluorescent because of the indole ring structure, and their fluorescence lifetime depends on the environment in which they are in. In buffer, most serotonin metabolites show a single exponential decay indicating a single fluorescence lifetime, as shown in Supporting Figure S1. The exception is tryptophan, which has two lifetimes (650 μs and 3 ns). Two new lifetime components around 650–1000 ps and 14–21 ns emerge in the presence of vesicles, indicating membrane binding. The relative amplitude of this lifetime can be used to estimate the fraction of serotonin metabolites bound to the membrane. We note that a few-component discrete model is inadequate to quantitatively analyze the Time-Correlated Single Photon Counting data arising from this rather heterogeneous specimen.^53^ Moreover, these data have been recorded only at a single concentration. Therefore, the quantities given here should be treated only as qualitative estimates. For tryptophan, both the amplitude of the ∼20 ns lifetime and the increase in the 650 ps lifetime component are used to calculate the lipid-bound serotonin metabolites. The exact lifetime and their amplitudes of all of the molecules can be found in Supporting Table S2.

**Figure 2:**
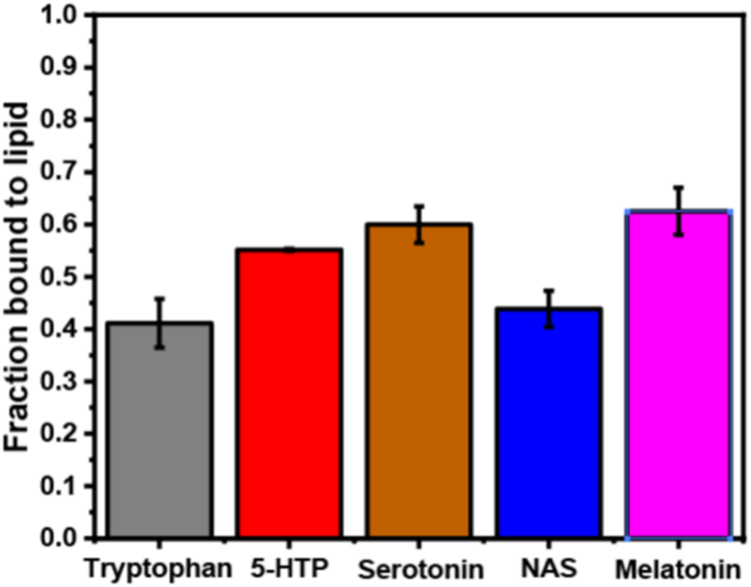
Fraction of bound serotonin metabolites to SUVs mimicking synaptic vesicles estimated using fluorescence lifetimes. The lipid composition of the SUVs was POPC/POPE/POPS/Chol (3/5/2/5 molar ratio) in 20 mM HEPES buffer (150 mM NaCl, pH 7.4).

While tryptophan and NAS show a lower membrane-bound fraction (around 40%), 5-HTP, serotonin, and melatonin exhibit a slightly higher binding (around 60%) as shown in Figure 2. Still, all serotonin metabolites show high membrane affinity, and therefore, they should all be considered as possible membrane modulators.

**Figure 3:**
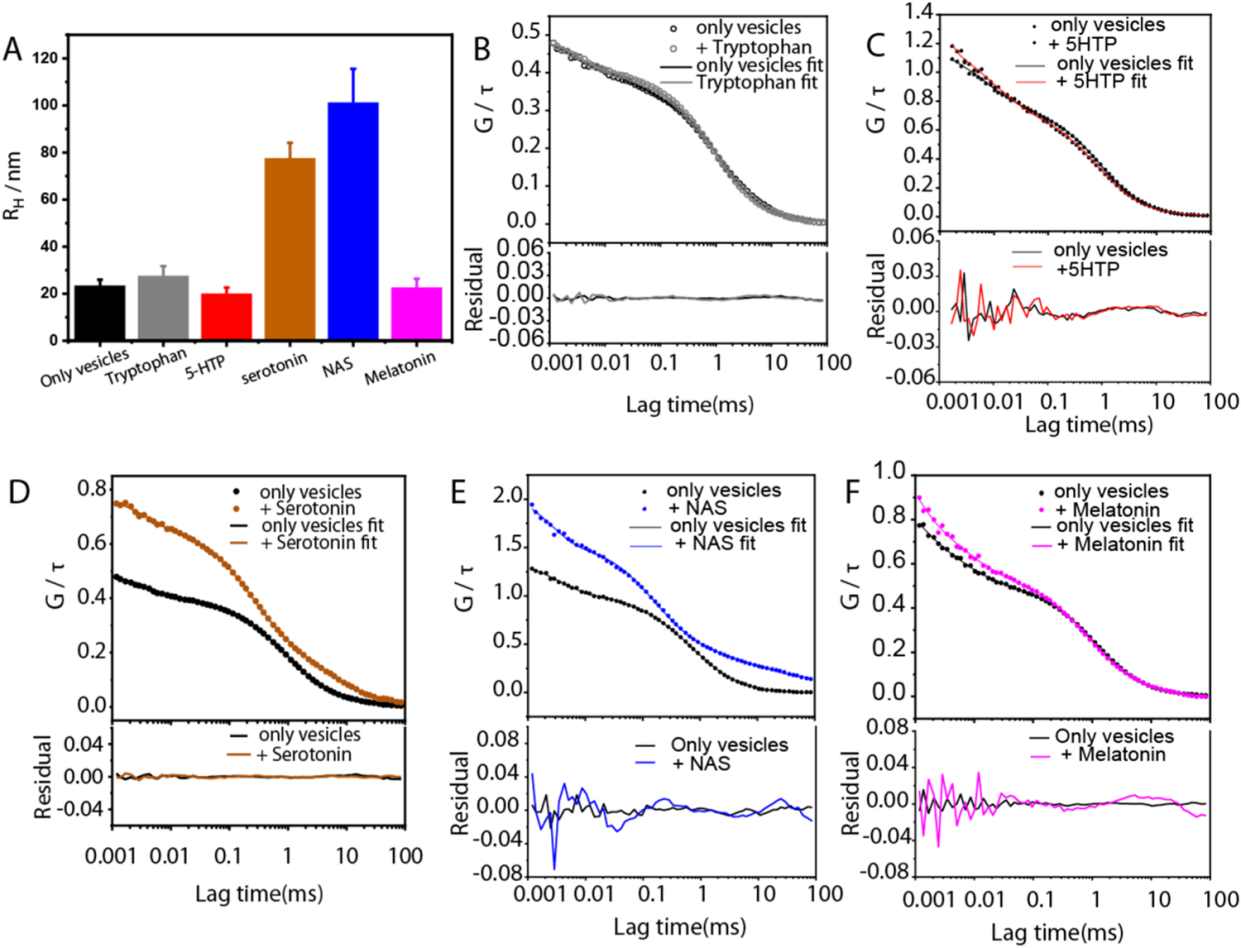
Measurement of vesicle association induced by serotonin metabolites in synaptic model membranes by FCS. Panel (A) shows the average hydrodynamic radii (*R*_H_) of SUVs in the absence and presence of 5 mM serotonin metabolites. Mean values ± SEM are plotted, *n* ≥ 4. Representative fluorescence autocorrelation curves of Nile red-labeled POPC/POPE/POPS/cholesterol, 3/5/2/5 molar ratio SUVs in 20 mM HEPES buffer (150 mM NaCl, pH 7.4) with their corresponding fits (solid lines) and residuals (solid lines at the bottom) in the absence and presence of 5 mM tryptophan (B), 5-HTP (C), serotonin (D), NAS (E), and melatonin (F).

All serotonin metabolites are found in the brain, and serotonin and melatonin are known to be active neurotransmitters.^27,54^ It is possible that the neurotransmitter could affect the association of vesicles, as has earlier been observed with serotonin.^15^ Here, we investigated whether increased vesicular association was observed for the other serotonin metabolites. We employed FCS of synaptic SUVs containing the fluorescence lipophilic label Nile red in the presence and absence of 5 mM different serotonin metabolites. FCS measures the diffusion of SUVs containing the fluorescent label Nile red. If the SUVs associate with each other, then the diffusion rate would slow down. This can be shown in the autocorrelation function (Figure 3B–F). To estimate an average hydrodynamic radius (*R*_H_), a three-dimensional model was used, containing two species to consider Nile red diffusion inside and outside vesicles, respectively. This fitted the FCS traces well, as the residuals are shown in Figure 3. The *R*_H_ changed significantly only for serotonin and its metabolite NAS (Figure 3A). This shows that the serotonin metabolites have wide but differing membrane modulation effects. While serotonin and NAS strongly promoted vesicle association, tryptophan, 5-hydroxytryptophan, and melatonin had no significant effects.

**Figure 4:**
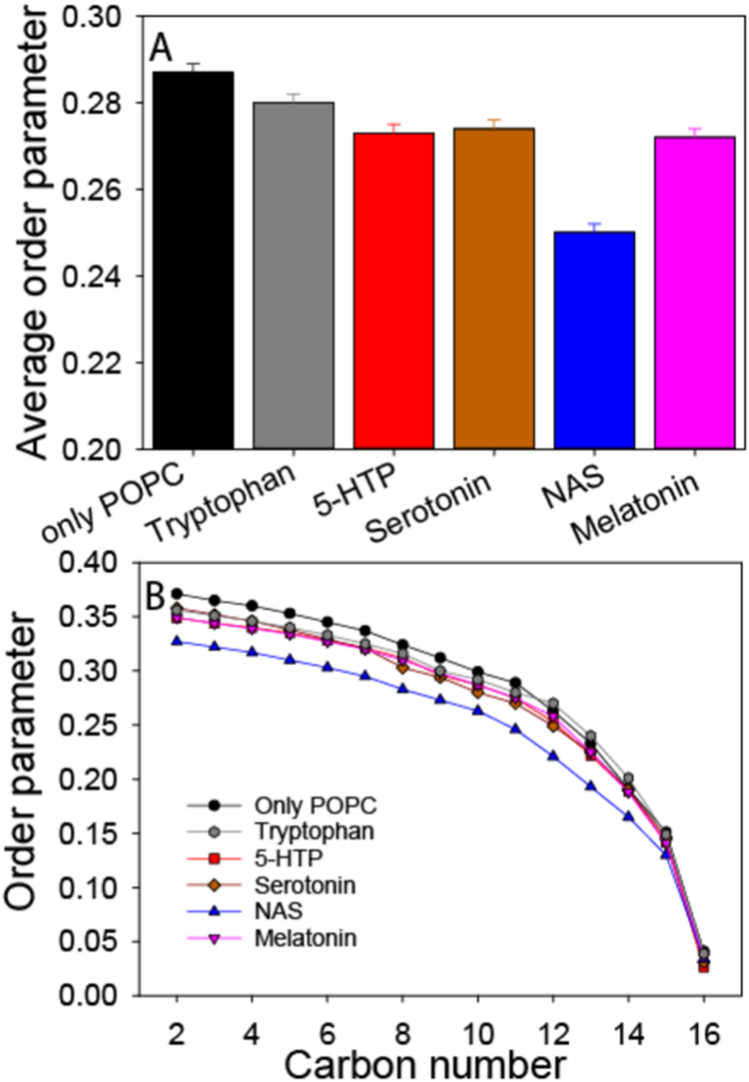
^2^H NMR average order parameters (A) and smoothed order parameter profiles (B) in a synaptic mimicking model membrane in the presence of 10 mol % serotonin metabolites. The synaptic model consisted of POPC-*d*_31_/POPE/POPS/Chol in the molar ratio 3/5/2/5 hydrated to 50 wt % using K_2_HPO_4_ buffer.

To explain these intriguing membrane modulating effects, we tested the impact of the serotonin metabolites on the acyl chain order of the synaptic membrane model using solid state ^2^H NMR spectroscopy. By deuterating the palmitoyl chain of POPC (POPC-*d*_31_) in vesicles with synaptic membrane-mimicking composition (POPC/POPE/POPS/cholesterol = 3/5/2/5 molar ratio), we could get an indication of how the serotonin metabolites modulate membrane properties. All of the ^2^H NMR spectra can be found in Supporting Figure S2. From the ^2^H NMR spectra, the chain order parameter was calculated.^55^ We observe that at a concentration of 10 mol %, all serotonin metabolites disordered the membranes but the degree of the disordering varied, when comparing the average chain order parameters (Figure 4). Tryptophan had the weakest effect, while serotonin, melatonin, and 5-HTP induced similar and more pronounced disordering. Interestingly, NAS had a much stronger effect than did the other neurotransmitters. Additionally, from the smoothed order parameter profiles, showing the order parameters for each individual carbon (Figure 4B), we compared the order changes with resolution along the lipids’ acyl chains. NAS lowers the order parameters for all of the carbons in the chain equally. In contrast, tryptophan decreases the order parameters for segments C2–C11. Serotonin also decreases the order parameters in the plateau region (C2–C6) while the lower half of the chain is not affected. For tryptophan, melatonin, and 5-HTP, a similar decrease in order is observed for the upper half, while the lower half of the chain is also somewhat disordered. These differences suggest that the average positions of the molecules vary in the membrane.

**Figure 5:**
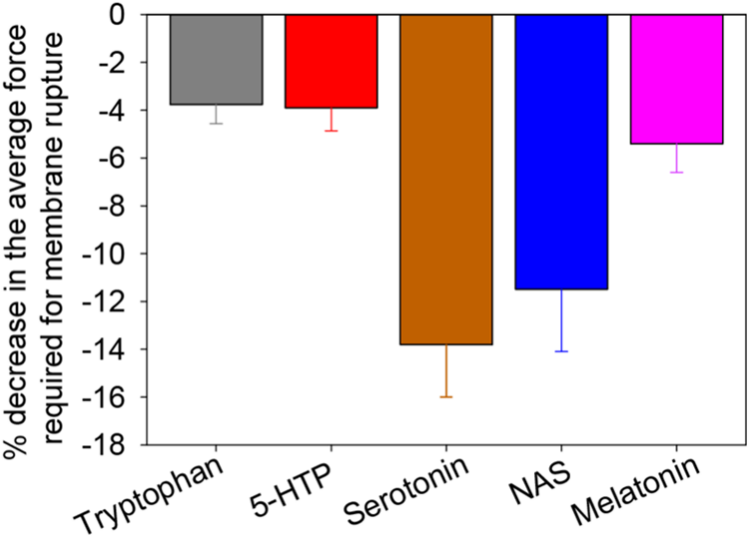
Fractional change in the AFM indentation force of supported synaptic membrane models in the presence of different serotonin metabolites. The plot shows the relative decrease in the average indentation force upon the addition of 20 mM of the individual serotonin metabolites. The supported lipid bilayer was composed of a 3/5/2/5 molar ratio of POPC/POPE/POPS/cholesterol hydrated in 20 mM HEPES buffer (150 mM NaCl, pH 7.4). The fractional change is calculated with respect to the control (no added molecule) for each bilayer, and then this quantity is averaged over bilayers (*n* = 3).

To test if the mechanical properties of the membrane, especially the resistance to indentation, change with added serotonin metabolites, AFM measurements were conducted on supported lipid bilayers mimicking synaptic vesicular membranes. The indentation force measured by using AFM is the force required to rupture a membrane and to form a pore. The force values correlate with the local stiffness of the membrane. We measured the indentation force for SLBs of synaptic vesicular composition at a 20 mM concentration of the individual serotonin metabolites. Figure 5 shows the % change in the average indentation force between the presence and absence of any small molecule. Histograms of the forces needed to rupture the membranes for the individual serotonin metabolites are shown in Supporting Figure S3. Here, we observe that serotonin shows maximum effect on the average indentation force closely followed by NAS. Other similar molecules like 5-HTP, tryptophan, and melatonin decrease the indentation force by only 4–5%. In agreement with lower order parameters, the decrease in average indentation force indicates a softer membrane due to the binding of the small molecules. The molecular-wise results correlate well with those observed for vesicle association.

**Figure 6:**
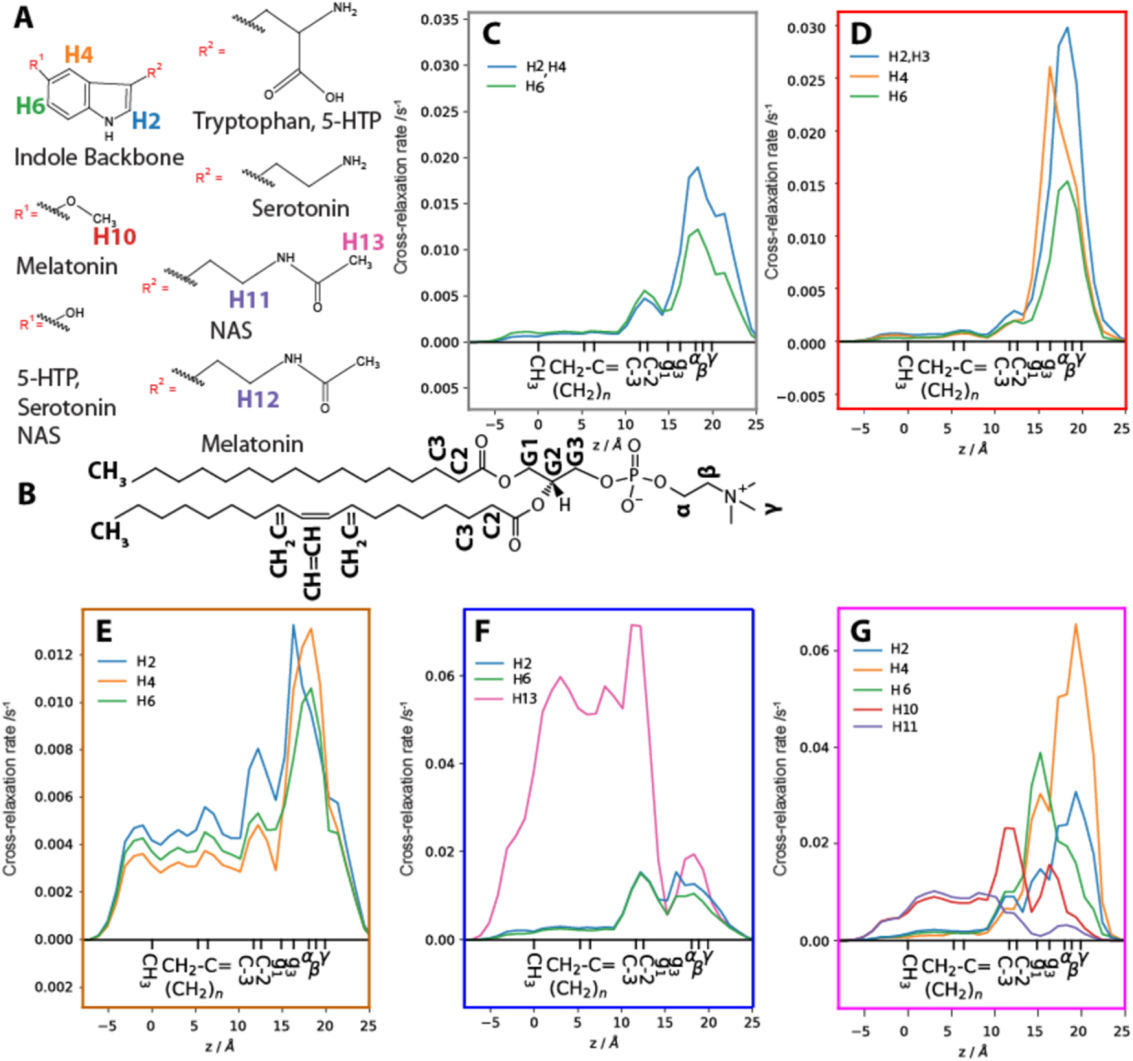
Membrane distributions of the serotonin metabolites in POPC membranes visualized by ^1^H MAS NMR NOESY. Protons selected from the molecules are shown for the common indole structure and structural modifications for the metabolites (A). Cross-relaxation rates between functional groups of POPC (B) and tryptophan (C), 5-HTP (D), Serotonin (E), NAS (F), and melatonin (G). The curves shown are obtained by multiplying the distribution of the selected lipid functional groups (CH_3_, CH_2_–C =, etc.) in the membrane, extracted from a previous MD simulation,^39^ by the NOE enhancement to that group, and then summing the resulting functions over all functional groups. The result is an estimate of the probability finding the selected atom at a given position in the membrane (see Figures S5–S9 for distributions prior to summation over the functional groups). The *x*-axis is set to zero at the mean position of the terminal CH_3_ group. The H number assignments follow the carbon numbering. The serotonin data are reproduced from^14^. The POPC membranes were hydrated to 50 wt % using K_2_HPO_4_ buffer.

The membrane distribution of the small molecules can differ even if they have a similar membrane affinity. Such alterations could explain why they interact differently with the membrane. To measure the distributions of the molecules in the membrane, we employed ^1^H NMR NOESY NMR under magic angle spinning (MAS) conditions for POPC membranes containing 20 mol % of the serotonin metabolites. The signals of the protons of the serotonin metabolites (Figure 6A) and the functional groups of POPC (Figure 6B) are well separated, allowing measurement of their intermolecular cross-relaxation rates (Figure 6B–G). These cross-relaxation rates provide a measure for the contact probability between molecular segments.^56^ To better quantify and visualize the distribution, how the molecules are distributed in the membrane, we extracted the distribution of the depth of POPC functional groups in the membrane from a previous MD simulation.^39^ Then, cross-relaxation rates from each lipid functional group to the serotonin metabolite ^1^H were multiplied by the corresponding distribution and added together, yielding an estimate of the probability of finding the ^1^H at each depth in the membrane (note that amplitudes are given in the units of the NOE transfer rate; therefore, this is not a true probability distribution). Zero on the *x*-axis corresponds to the mean position of the lipid methyl groups roughly corresponding to the midplane of the membrane and is labeled both in angstroms and marked with mean positions of each lipid functional group. Selected protons of the serotonin metabolites corresponding to several sides of the indole rings are shown in Figure 6, including some protons for the individual side chains when available. All protons of the serotonin metabolites that produced cross peaks are shown in Supporting Figures S4–S9.

For tryptophan, a broad distribution function of protons H2, H4, and H6 of the indole ring with two maxima is obtained, one minor one around C3–C2 and the major in the glycerol/headgroup region (Figure 6C). This indicates that tryptophan is distributed mostly in the lipid–water interface and in the headgroup region of the membrane. The indole ring of 5-HTP is predominantly found in the glycerol and headgroup region in a narrower distribution than that of tryptophan (Figure 6D). The differences between the individual protons of 5-HTP indole ring are also small. This indicates a relatively shallow insertion of the molecule with a rather homogeneous distribution. The indole ring of serotonin is more widely distributed with a clear preference for the water interface glycerol and headgroup region but is also present further down the chain (Figure 6E). This suggests a more evenly distributed serotonin in the membrane. The indole ring protons H6 and H2 for NAS are found in two populations, one along the C2–C3 and one in the interface region (Figure 6E). Interestingly, the end of the side chain (H13) is found along the entire chain region of POPC. In addition, the H10 proton found (Supporting Figures S4C and S5) also indicates that it is localized somewhat deeper than the rest of the indole ring, possibly explaining the lower order parameter found for the NAS containing membranes (Figure 4). For melatonin, we found a heterogeneous distribution of each proton (Figure 6G). The indole ring with protons H2, H6, and H4 seems to be distributed at two populations either in glycerol or in the headgroup region. Interestingly, the lack of a polar OH group could possibly lead to a broader distribution because no hydrogen bond formation is possible. However, we detect cross relaxation from the methoxy group, and it has two populations, one stronger around C2–C3, one weaker around the glycerol region, and a broad smaller component further down the chain. This suggests that the methoxy group dips slightly down in the membrane. In addition, H12 in the side chain is mostly distributed in the acyl chain region. This suggests that the side chain is at least partially buried in the membrane. The lack of an OH group in the indole ring seems to lead to a broader distribution for melatonin.

To further understand the membrane distribution of the serotonin metabolites, we employed MD simulations. In NOESY NMR, we are limited to POPC only membranes because the chemical shifts of the different phospholipids would superimpose which impairs analysis of cross-relaxation rates. Furthermore, cholesterol adds significantly to the spectral complexity and broadens the NMR signals, yielding insufficient spectral quality. In the MD simulations, we could use a synaptic model membrane mimic at physiological temperatures as was used in the other experiments (POPC/POPE/POPS/cholesterol 3/5/2/5 molar ratio). In addition, MD provides other useful information about the membranes like H-bonds between serotonin metabolites and the membrane.

**Figure 7:**
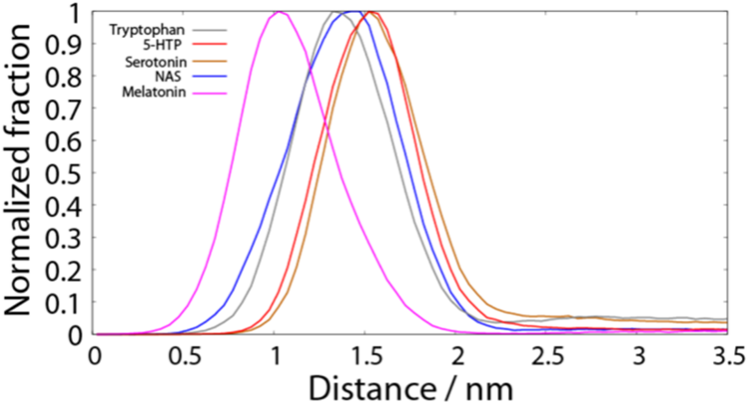
Normalized fraction of neurotransmitters, calculated as the cumulative occurrence of 20 molecules at a given distance from the MD simulations. This distance is defined as the lowest distance between any heavy atom of each of the 20 neurotransmitters and the center of the lipid bilayer simulated at 37 °C. The normalization was performed by dividing the population of each bin (0.05 nm) by the population of the most populated bin, calculated separately for each molecule. The simulated lipid bilayer was composed of a 3/5/2/5 molar ratio of POPC/POPE/POPS/cholesterol.

The simulations revealed that the addition of a low concentration of neurotransmitters (2 molecules per 240 lipids, ∼0.8%) had a minimal impact on the lipid bilayer. In contrast, a higher concentration (20 molecules per 240 lipids, ∼8%) resulted in noticeable effects, as also seen by other methods. Serotonin, the only charged neurotransmitter in the study, penetrated the lipid bilayer least, residing mainly in the hydrophilic part of the membrane (Figure 7). This somewhat contrasts with the results obtained for the zwitterionic POPC membranes, where they were more widely distributed (Figure 6). Possibly, Coulombic forces with the negative surface charge favorably stabilize the molecules near the phosphate and amide groups of the PS molecules. On the other hand, the charge state of serotonin at the membrane surface is unclear but may vary depending on the complicated surface structure of lipid membranes. In contrast, neutral neurotransmitters penetrate deeper into the bilayer with melatonin exhibiting the highest affinity and the deepest embedding into the lipid bilayer, followed by NAS. Especially, the side chain of these molecules could form a hydrogen bond between its NH group and the CO of the acyl chains, allowing for the hydrophobic end of the side chain to insert deeper in the membrane.

Tryptophan, 5-HTP and serotonin itself position themselves in a very similar manner with respect to the lipid bilayer, with the C5 atom being deepest, then N1 and CA being shallowest in the lipid bilayer (Figures S10 and S11). This pattern changes completely upon acetylation (NAS), when all of these parts are positioned at similar distances from the lipid bilayer center. Addition of the methyl group to NAS results in a strong tendency of melatonin to anchor deeply in the lipid bilayer, while other parts of the compound remained at similar depths. This positioning is reflected in the NOESY data (Figure 6), where melatonin shows a heterogeneous distribution with its methoxy group dipping deeper into the membrane compared to other metabolites.

**Figure 8:**
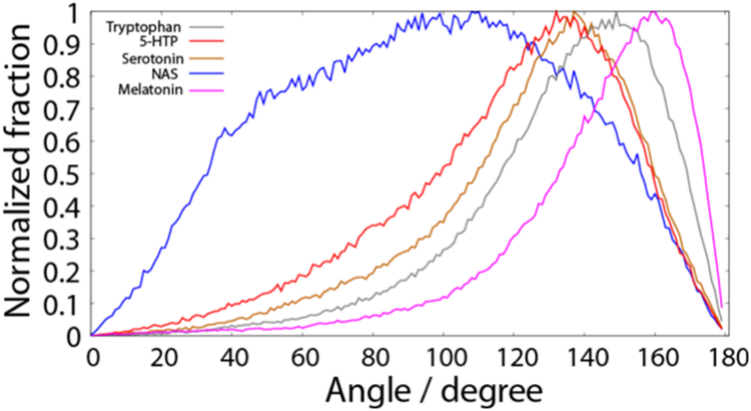
Orientational distribution of the neurotransmitters in relation to the lipid bilayer calculated from MD simulation. The angle is calculated between the vector formed by the C2 and C5–C6 atoms of the almost-rigid indole ring, present in all studied neurotransmitters, and the *z*-axis, which is also normal to the lipid bilayer, as in our previous work.^37^ The simulated lipid bilayer was composed of a 3/5/2/5 molar ratio of POPC/POPE/POPS/cholesterol.

In all examined molecules, there are two protonated nitrogen atoms able to form H- bonds: one in the indole ring (N1) and one in the amine group position (N) (Figure S10). We focused on these two atoms because they are found in all serotonin metabolites. We included both H-bond donors and H-acceptors. While the direct surrounding of the nitrogen in the indole ring remains unchanged, its tendency to form hydrogen bonds with hydrophilic parts of the lipids depends on the general orientation and position of the whole molecule rather than local surroundings. The tendency of N1 to form hydrogen bonds is somewhat larger for 5-HTP and serotonin, consistent with their tendency to stay shallower in the lipid bilayer compared to other molecules (Table S3). The N atom’s tendency to form hydrogen bonds is much more diverse, being lowest for tryptophan and highest for serotonin and 5-HTP. This is caused by the addition of the hydroxyl group to the indole ring, which tilts the molecule and makes it more preferable to stay closer to the lipid–water interface (Figure 8). For NAS and melatonin, this effect is modified by the changed hydrophobicity of these derivatives, which causes NAS to wobble and melatonin to position specifically inside the hydrophobic part of the lipid bilayer.

The presence of neurotransmitters also decreased the number of water molecules in the interface of the bilayer (∼1.7–2.1 nm from the lipid bilayer center) due to the presence of the serotonin metabolites mostly in the hydrophilic part of the membrane, which limited the space available for water molecules to enter (Supporting Figure S12). However, the presence of all ligands increased the number of water molecules reaching the hydrophobic part, with the effect being most pronounced for tryptophan and least for serotonin. This differential impact on water distribution likely reflects the distinct membrane localization patterns of these metabolites and their ability to create water-permissive pathways through the membrane. The increased water penetration might also be related to the membrane disordering effects observed in the NMR experiments, as more disordered lipid packing could allow greater water access to the hydrophobic core.

Interestingly, the presence of the neurotransmitters only marginally increased the surface of the lipid bilayer in MD simulations (Supporting Table S3). This effect was smallest for the serotonin (0.5%) and largest for NAS (0.6%) and melatonin (0.7%). This effect may be connected to the distinct behavior of NAS compared to other studied neurotransmitters (Figure 8), as it is the only molecule that does not have a strong preference for maintaining a near-parallel orientation of the indole ring to the lipid bilayer normal but rather wobbles considerably with a slight preference for orienting its indole ring perpendicular to the lipid bilayer normal. Most of the studied neurotransmitters resided for the majority of the simulation time at the dist_min_ of 1.5 nm (5-HPT and serotonin) and 1.4 nm (tryptophan and NAS). Taking into account the total length of the neurotransmitters in the range of 0.8–1.0 nm, this suggests that they are located in the hydrophilic part of the lipid membrane. Only melatonin penetrates deeper into the upper acyl chain region, with the peak of the minimum distance to the lipid bilayer center around 1.0 nm. This is visible in the high degree of solvent-accessible surface area (SASA) hidden from the solvent (buried SASA) for melatonin (Table S3); however, this value is even larger for serotonin, despite it being shallower in the lipid bilayer. This results from the fact that among the studied neurotransmitters, serotonin is the smallest molecule, while melatonin is the largest, with its methoxy group attached to the indole group penetrating the lipid bilayer deeper than other molecules without modifications in this position (Figure 1), as was also observed in the NOESY data (Figure 6). Interestingly, the NAS shows the third-highest buried SASA value, despite its shallower and more unstable mode of penetration.

**Figure 9:**
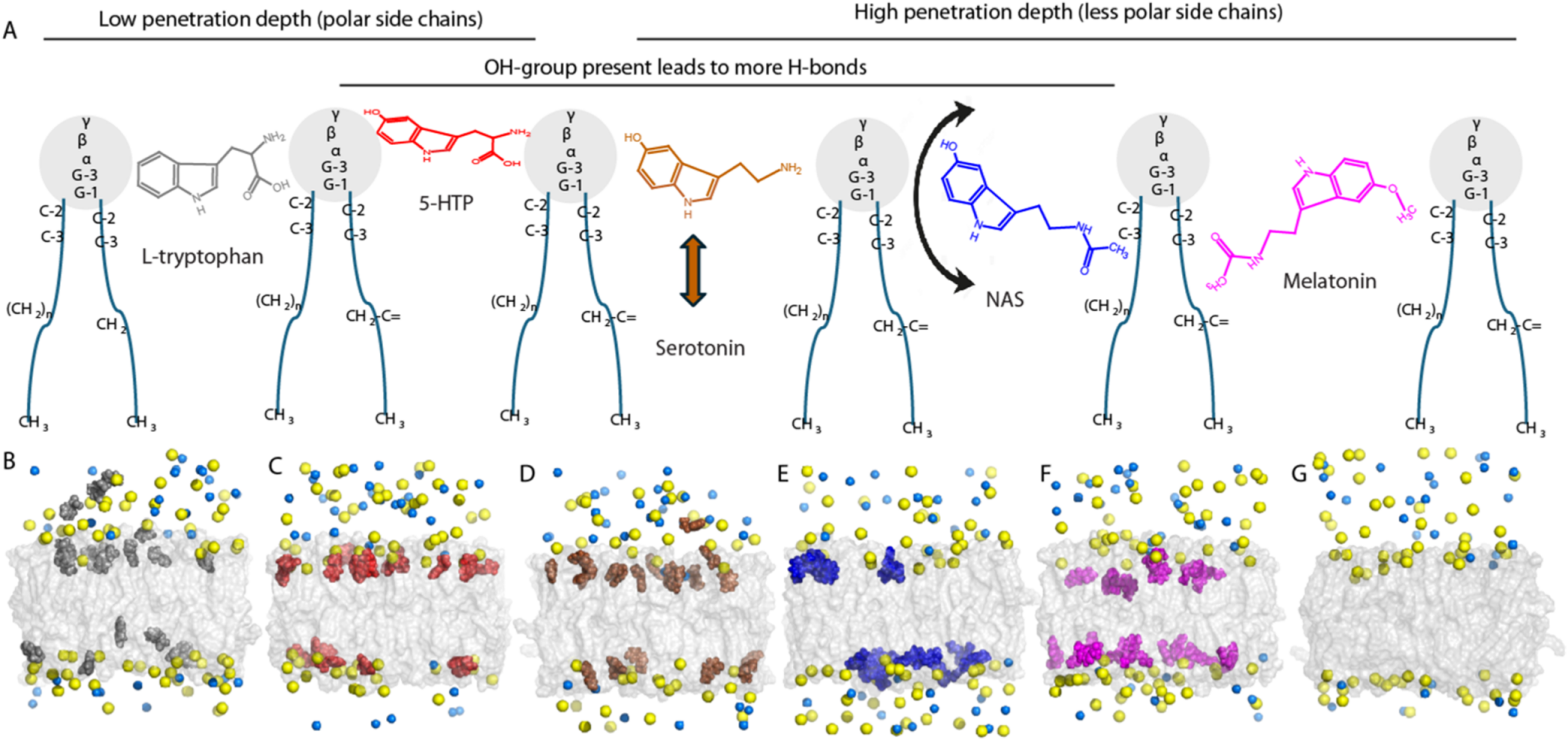
Suggested schematic representation of the serotonin metabolite distribution in membranes. Panel (A) shows suggestive distribution of the serotonin metabolites in POPC membranes based on the NOESY data. Arrows indicate large movements of the molecules. Panels (B–G) show suggestive distribution of the serotonin metabolites in the synaptic mimicking membranes in the MD simulations. (B) Tryptophan, (C) 5-HTP, (D) serotonin, (E) NAS, (F) melatonin, and (G) only water. The lipid bilayer is presented as a gray surface, serotonin metabolites in respective color code, while yellow and dark blue spheres represent Na^+^ and Cl^–^ ions, respectively. Water is not shown to maintain the clarity of the figure.

In summary, all serotonin metabolites modulated the membrane but to a different degree. The membrane distribution of the serotonin metabolites also differed, as observed in both the NOESY results in POPC membranes and for the synaptic mimicking membranes used in the MD. A suggested summary of the results is shown in Figure 9.

## Discussion

We previously reported that serotonin had numerous effects on the physical properties of lipid membranes^6,14,37^ and even influenced the function of a membrane receptor that has no reported relation to serotonin.^8^ Based on these findings, the aim of the current study was to investigate if a family of molecules relevant for an important metabolic pathway starting with tryptophan also induces such membrane modulation effects and, if so, whether these effects differ in magnitude for the respective molecules. In general, our results suggest a complex but rather specific interaction profile between these components, influencing the bilayer structure, dynamics, and function, while the different penetration depth of these neurotransmitters into the lipid bilayer may have implications for their biological activity and interaction with membrane-bound proteins.

All molecules studied here influence membrane properties, but the degree to which they do it varies. Structurally, all of the metabolites are based on the indole ring but feature small modifications. Indole rings are known for their high membrane affinity and localization in the lipid water interface of the membrane.^57^ Various physical interactions are responsible for the membrane affinity, while the exact degree of binding and penetration depth is subject to the individual lowest free energy of the system, also related to the exact composition of the bilayer. Our results also highlight that the membrane localization of the neurotransmitters could be important to hinder or enhance membrane modulating effects, e.g., by modulating vesicular fusion^15^ or membrane protein activity, e.g., membrane receptor activation.^20^ Possibly, molecules that interfere with the sequestration of neurotransmitters, e.g., SSRI, cocaine, or amphetamine, could then alter the membrane modulation that is normally fine-tuned by nature. To examine the membrane modulating effects of the serotonin metabolites, we explored binding affinity, effects on the acyl chain order, and membrane distribution of the different serotonin metabolites.

We found tryptophan to have the least effect on membrane fluidity (Figure 4), possibly suggesting that it is just an intermediate in membrane modulating neurotransmitters. This makes sense because tryptophan is an essential amino acid and therefore it is broadly distributed in the body. In contrast, the neurotransmitters, especially NAS and melatonin, are narrowly distributed in the body.^58^ Therefore, it seems that molecules having more membrane modulation are regulated to be more carefully distributed in the body. The tryptophan residues of membrane proteins have earlier been reported to often reside in the membrane water interface,^57,59^ agreeing with our results (Figures 6, 7 and 9). Model transmembrane peptides like WALP were even designed to use tryptophan to anchor the proteins to the membrane water interface,^60^ suggesting that this specific location of tryptophan anchors proteins within the membrane. This interfacial localization is further supported by MD simulations showing that tryptophan forms few hydrogen bonds with the lipid bilayer, maintains a relatively stable orientation nearly parallel to the lipid bilayer normal, and has a minimal impact on lipid fluidity. However, by variations in the side chain as observed for the other serotonin metabolites (Figure 6), the membrane distribution changes.

The effect of serotonin on membrane properties is by far the most studied. We previously found that serotonin modulates both physical parameters, e.g., lipid chain order parameter and membrane stiffness, as well as biological effects such as membrane association, ligand binding, and lipid domain sizes.^6,8,14,15^ We observed that serotonin had the largest effect on vesicle association (Figure 3). For decreasing acyl chain order in the membrane, serotonin showed a similar effect as 5-HTP and melatonin (Figure 4). Serotonin is known to disorder membranes,^8^ but synaptic vesicles were shown to tolerate unusually high concentrations of serotonin before they get disordered.^37^ Possibly the smaller size of serotonin compared to that of NAS leads to a smaller extent of disruption, even if both molecules were also found deeper in the membrane (Figure 6). However, the mechanical properties measured in the AFM experiments seemed to be similar for serotonin and NAS (Figure 5). Both the order parameters and the NOESY data indicate that serotonin would be found in both the membrane water interface and deeper down in the membrane (Figures 4 and 6).

5-HTP was found to reside shallowly in the membrane in both the NOESY and MD simulations (Figures 6 and 8). The introduction of the OH group lead to more H-bonds (1.43 per molecule vs 0.72 for tryptophan and tryptophan) because of the change tilt of the molecule observed in the MD simulations (Supporting Table S3).

NAS has been shown to bind to the melatonin receptor.^61^ However, NAS has also been found in brain areas not associated with melatonin synthesis.^62^ NAS but not melatonin affects tropomyosin receptor kinase B (TrkB) signaling possibly leading to melatonin independent antidepressant and circadian rhythm effects in the brain.^63^ However, in a binding assay for orthosteric agonists for TrKB, no orthosteric binding was found for NAS.^64^Therefore, the strong membrane modulating effect we observed could possibly play a role. We found that NAS had a very strong effect on the lipid order parameter (Figure 4). This could be related to the side chain protruding deep into the membrane, as supported by ^1^H MAS NOESY NMR (Figure 6), therefore leading to increased membrane fluidity. In addition, in the MD simulations, it was found that it wobbled significantly in the membrane (Figure 8), possibly also leading to lower order parameters. The high membrane disruption was also observed in the AFM experiments (Figure 5), possibly related to the low order parameters (Figure 4) and the wobbling of NAS (Figure 8). Interestingly, NAS also affected the vesicle diffusion in the FCS measurements (Figure 3), indicating that it could increase vesicle aggregation. This strong membrane modulation and its effect on cell signaling is probably the reason that is very narrowly distributed in the brain.

There is some lack of correspondence between the membrane distribution of the serotonin metabolites as indicated by MD simulation and that found by NMR. Several possibilities like the difference between POPC and synaptic membranes or small differences in concentration could affect. Another possible reason for this is the unknown charge state of the molecule near and inside the membrane. It was observed in the quantum mechanical calculations that the dipole moment for the serotonin metabolites was quite different in the gaseous (resembles the membranes) and the aqueous phase (similar to the water phase) as shown in Figure 1 and Table S1. This also suggests that the distribution can be a very strong function of the local pH. This should serve as a cautionary tale for similar efforts to deduce the location of small amphiphiles in the membrane.

Melatonin has several functions, including regulating circadian rhythm and sleep.^27^ Both NAS and melatonin have been proposed to act as antioxidants by scavenging free radical oxygen species,^65^ possibly requiring different membrane distribution than the other metabolites. While melatonin has several receptors, it has also been proposed to modulate membranes in earlier research.^29,66^ Using neutron scattering, it was found that melatonin decreased membrane thickness in 1,2-dioleoyl-*sn*-glycero-3-phosphocholine (DOPC) and 1,2-dipalmitoyl-*sn*-glycero-3-phosphocholine (DPPC) in the presence and absence of cholesterol,^66^ in agreement with our observations (Figure 4). MD simulations in DPPC and DOPC membranes showed melatonin resided in the membrane interface.^66^ Melatonin had a very deep distribution in the synaptic membranes in our MD simulation (Figure 8) and a very broad distribution in the POPC membranes (Figure 6). The lack of the OH group in melatonin leads to the lowest amount of H-bonds (0.39 per molecule) of all of the serotonin metabolites (Supporting Table S3). By having fewer interactions with neighboring lipids or other melatonin molecules, this could lead to melatonin being distributed deeper (Figure 8) or more widely in the membranes than other serotonin metabolites.

## Conclusions

In summary, we found that all serotonin metabolites modulated the membrane to some degree, but some had stronger effects than others. Removing the carboxyl group of the side chain of the serotonin metabolites led to a deeper distribution of the small molecules in the POPC membranes. However, some effect could clearly be membrane-specific, e.g., the charged PS could hinder a charged serotonin to reach deeper in the membrane as observed in the MD simulations, so lipid-specific effects need to be considered. The addition of an OH-group to the indole aromatic ring led to stronger H-bond formation, anchoring the molecules more to the water interface. While individual modifications of the molecular structure lead to differences in membrane modulation and distribution, a straightforward prediction and correlation of structure and membrane modulation remains difficult. It is clear that not only serotonin but also some of its metabolites exhibit strong membrane modulating properties. Therefore, all possible mechanisms to modulate membrane properties need to be considered for understanding all modes of action of neurotransmitters and small-molecule drugs. In a longer perspective, developing molecules with specific membrane modulation effects that influence receptor activation may be relevant for new drug development.

## Supporting Information Available

Fluorescence and NMR spectra, AFM histograms, COESY cross-relaxation rates, additional MD analysis graphs and tables with parameters from quantum calculations, fluorescence lifetime analysis, and MD parameters (PDF)
PDB files (ZIP)
