# Supplementary material for: Molecules in the Serotonin-Melatonin Synthesis Pathway Have Distinct Interactions with Lipid Membranes
Source: J Phys Chem B. 2025 Feb 28;129(10):2687–700. doi: 10.1021/acs.jpcb.4c08750 (PMC11912468; doi:10.1021/acs.jpcb.4c08750)
Supplement: Supplementary file 1 — jp4c08750_si_001.pdf [file jp4c08750_si_001.pdf]

The Molecules in the Serotonin-Melatonin Synthesis Pathway Have Distinct Interactions with Lipid Membranes

Oskar Engberg#[1], Debsankar Saha Roy# [2], Pawel Krupa[3], Shankha Banerjee[2], Ankur Chaudhary[2], Albert A. Smith[1], Mai Suan Li[34], Sudipta Maiti[2,5], Daniel Huster[1]

[1]Institute of Medical Physics and Biophysics, Medical Department, Leipzig University, Härtelstr. 16-18, D-04107 Leipzig, Germany.

[2] Department of Chemical Sciences, Tata Institute of Fundamental Research, Homi Bhabha Road, Colaba, Mumbai 400005, India.

3 Institute of Physics, Polish Academy of Sciences, Warsaw 02-668, Poland

4 Institute for Computational Science and Technology, Quang Trung Software City, Tan Chanh Hiep Ward, District 12, 729110 Ho Chi Minh City, Vietnam

5 Current address: Dept. of Biological Sciences and Dept. of Physics, Birla Institute of Technology and Science Pilani (BITS-Pilani), Hyderabad Campus, Shamirpet, Hyderabad 400078, INDIA

# = equal contribution

Correspondence: daniel.huster@medizin.uni-leipzig.de

sudipta.maiti@hyderabad.bits-pilani.ac.in

Supporting information

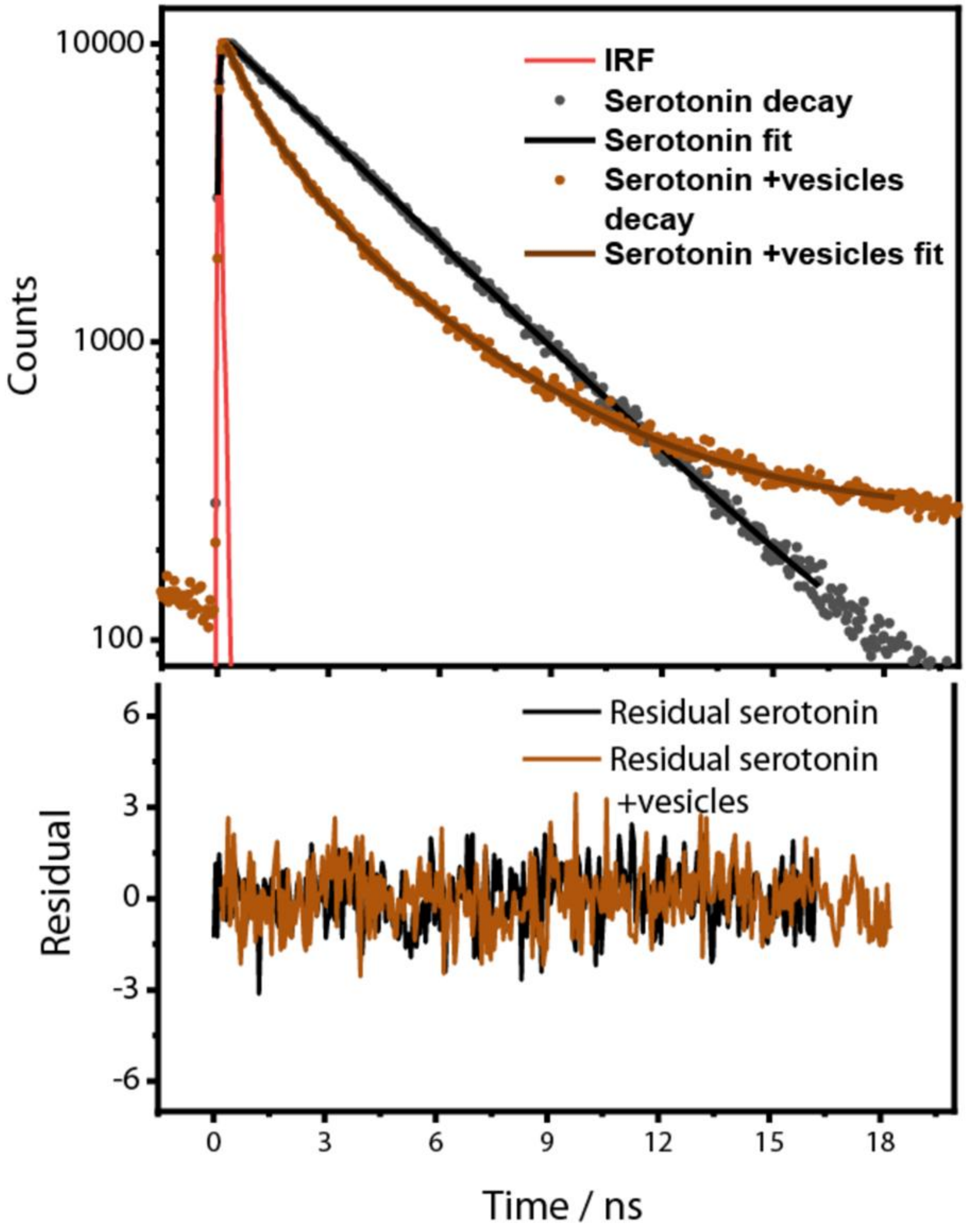

Figure S1. Representative fluorescence exponential decay of serotonin in the absence and presence of SUVs mimicking synaptic vesicles. In the presence of 10 mg/ml SUVs, serotonin shows multi exponential decay. The SUVs consisted of POPC/POPE/POPS/Chol in the molar ratio 3/5/2/5.

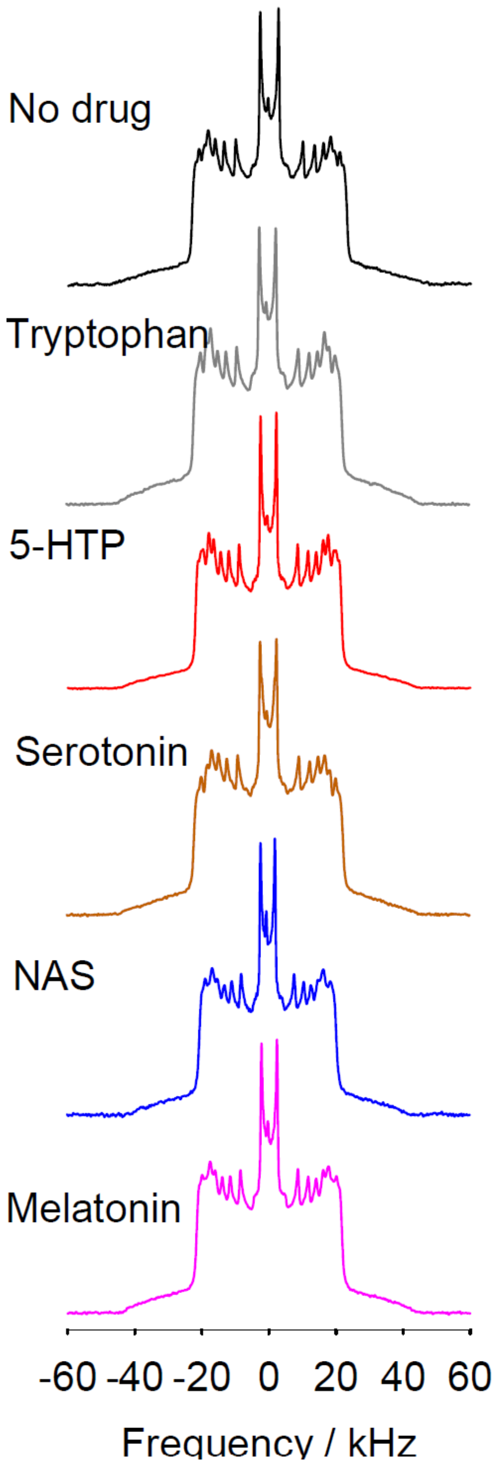

Figure S2. ^{2}H NMR spectra of synaptic mimicking MLVs in the presence and absence of 10 mol% serotonin metabolites. The synaptic model consisted of POPC-d_{31}/POPE/POPS/Chol in the molar ratio 3/5/2/5 hydrated to 50 wt% using a K_2HPO_4 buffer.

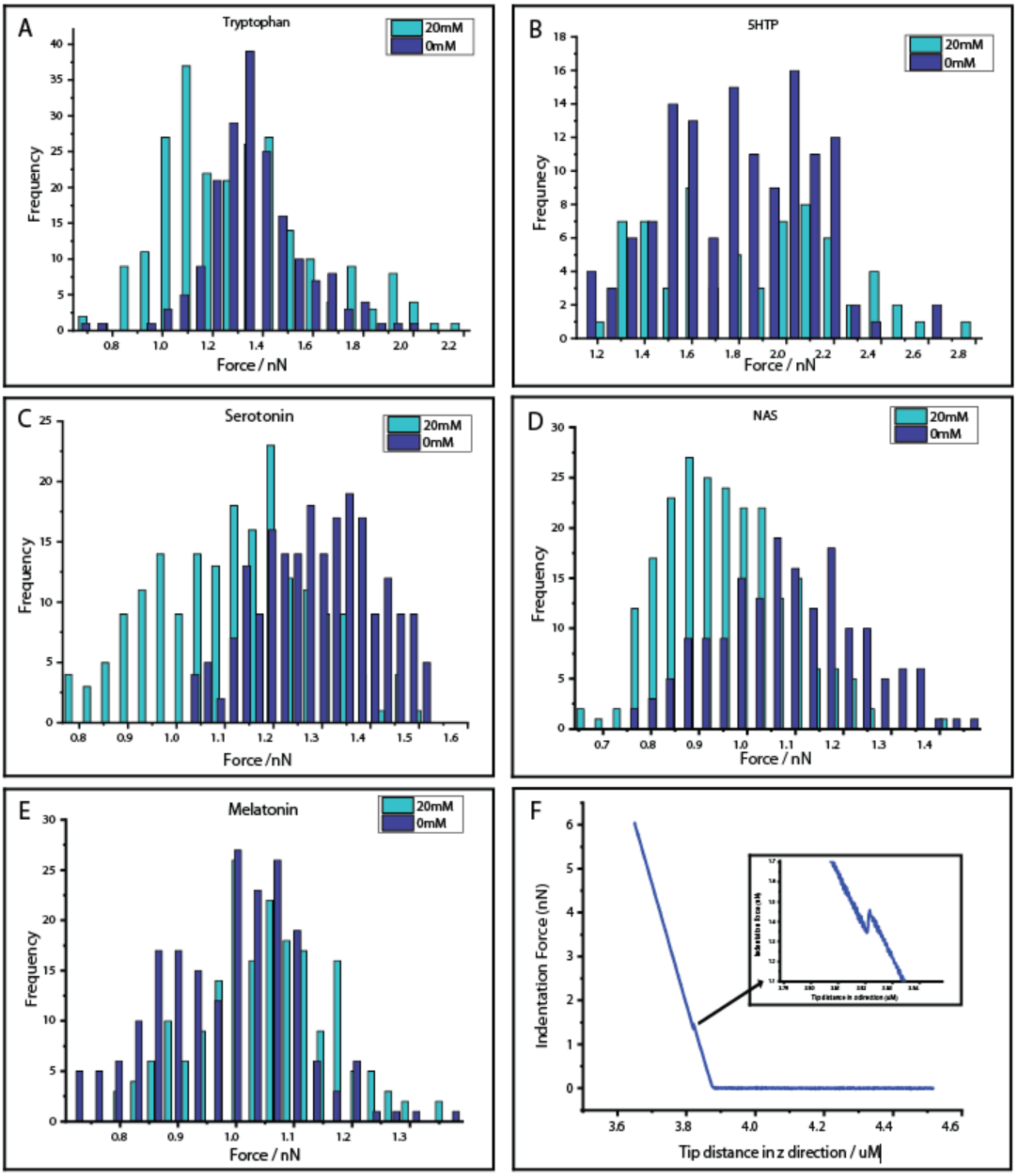

Figure S3. Measuring the fractional change in the AFM indentation force upon adding different serotonin metabolites in synaptic vesicle membrane mimicking supporting lipid bilayers. Panel A-E shows distribution of indentation forces as histograms in the presence and absence of 20 mM of the serotonin metabolites on a supported lipid bilayer of composition 3/5/2/5 molar ratio (POPC/POPE/POPS/Cholesterol). Panel F shows representative AFM indentation force measurement, inset shows the breakthrough point where the tip breaks the bilayer.

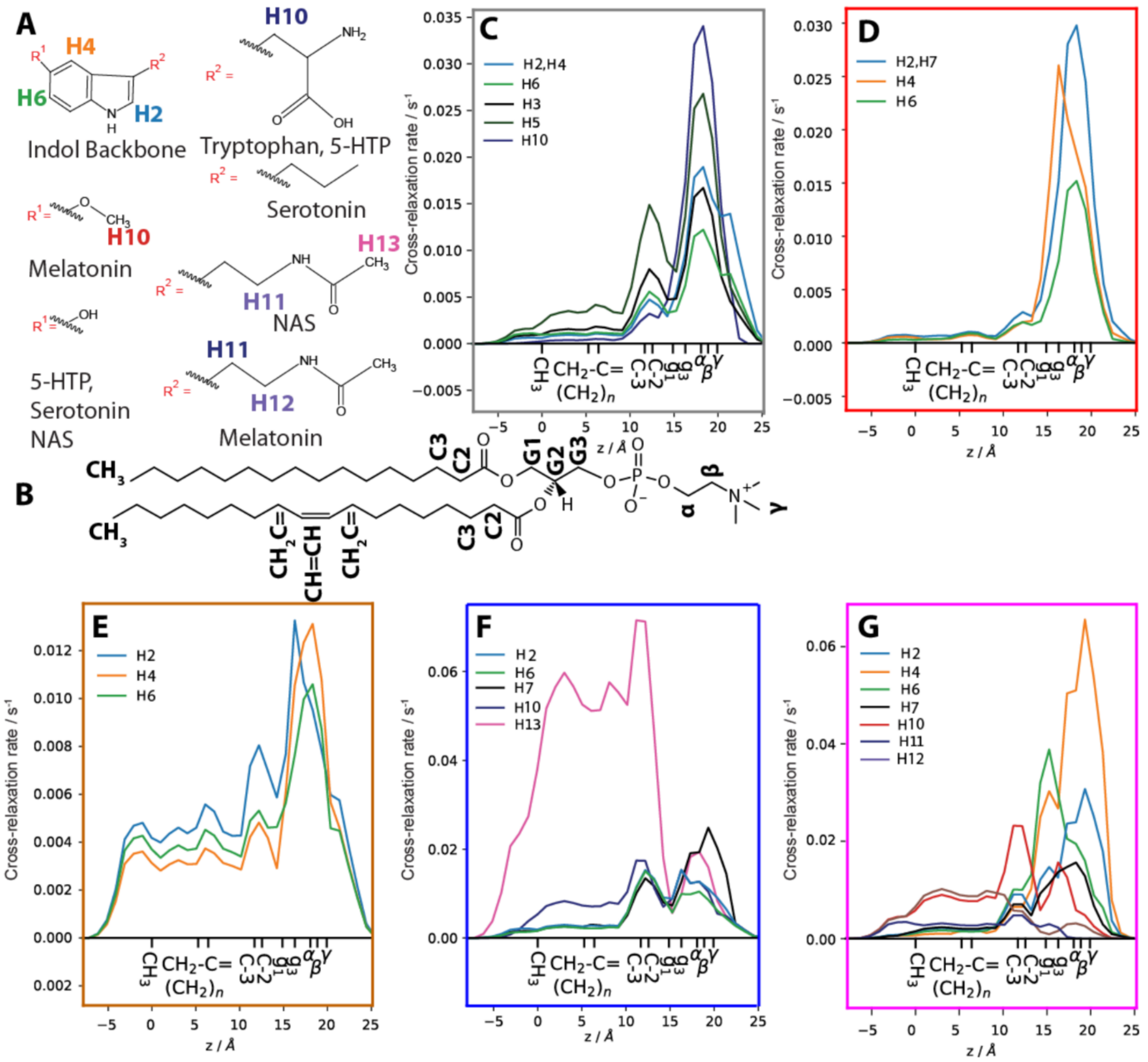

Figure S4: Membrane distributions of 20 mol% serotonin metabolites in POPC as measured by ^{1}H NMR NOESY NMR under MAS conditions. All measured protons are shown for the common indole structure and for the structural modifications for the metabolites (A). Cross-relaxation rates between functional groups of POPC (B) and tryptophan (C), 5-HTP (D), Ser (E), NAS (F), and Melatonin (G) are shown. The curves shown are obtained by multiplying the distribution of the selected lipid functional groups (CH_3, CH_2-C=, etc.) in the membrane, extracted from MD simulation, by the NOE enhancement to that group, and then summing the resulting functions over all functional groups. The result is an estimate of the probability finding the selected atom at a given position in the membrane. The x-axis is set to zero at the mean position of the terminal CH_3 group. Certain cross relaxation rates were excluded because of intramolecular interactions. The H number assaigments follow the carbon numbering. The POPC membranes were hydrated to 50 wt% using K_2HPO_4 buffer. The serotonin data is reproduced from[14].

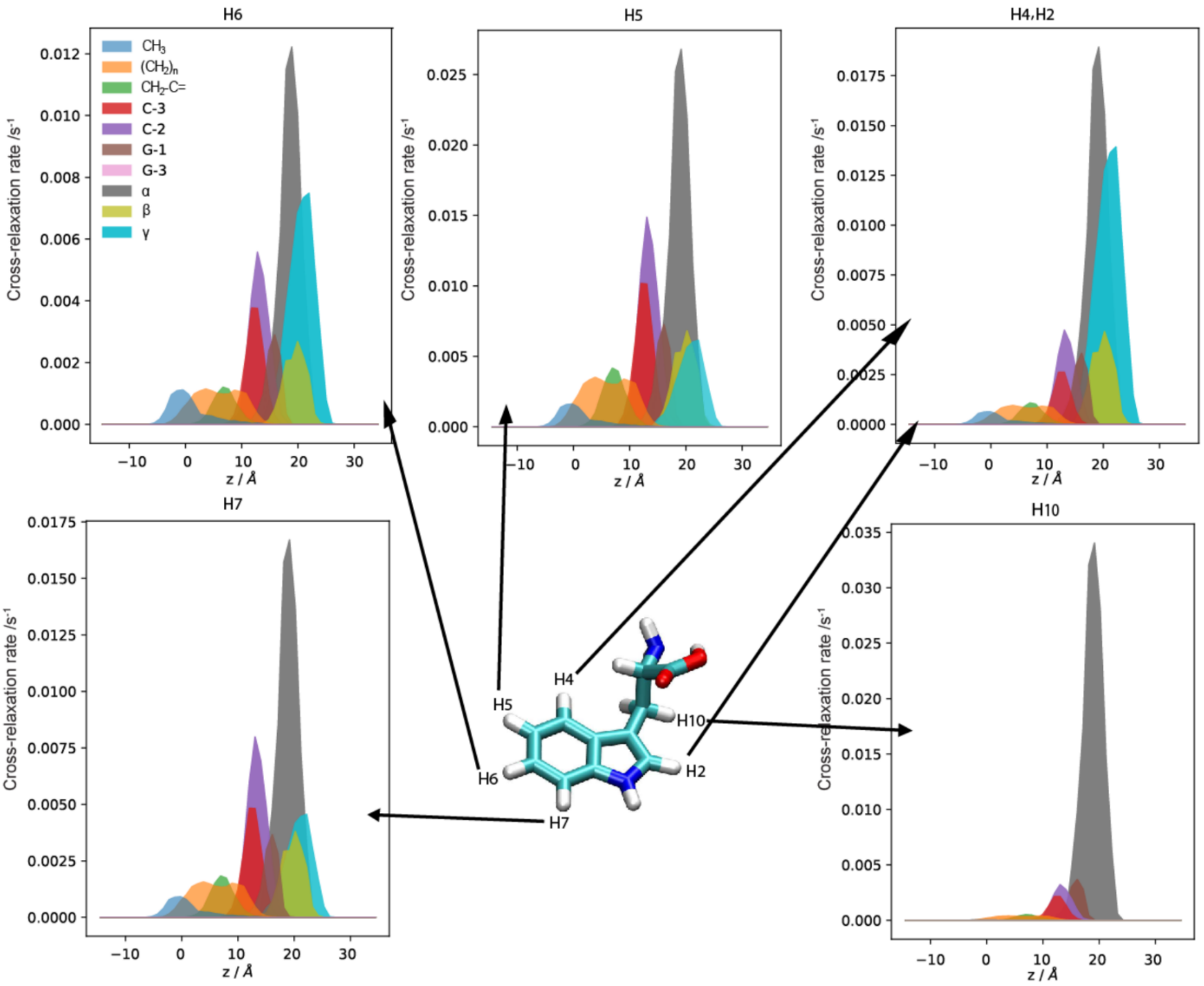

Figure S5. Membrane distributions of 20 mol% tryptophan in POPC as measured by ^{1}H NMR NOESY NMR under MAS conditions. Shown for all protons measured. For each NOE rate measured, we display the depth distribution of the given functional group on POPC, and scale that distribution by the measured NOE rate constant. Zero on the x-axis corresponds to the mean position of the lipid methyl groups. Certain NOEs were excluded because of intramolecular interactions. The POPC membranes were hydrated to 50 wt% using K_2HPO_4 buffer.

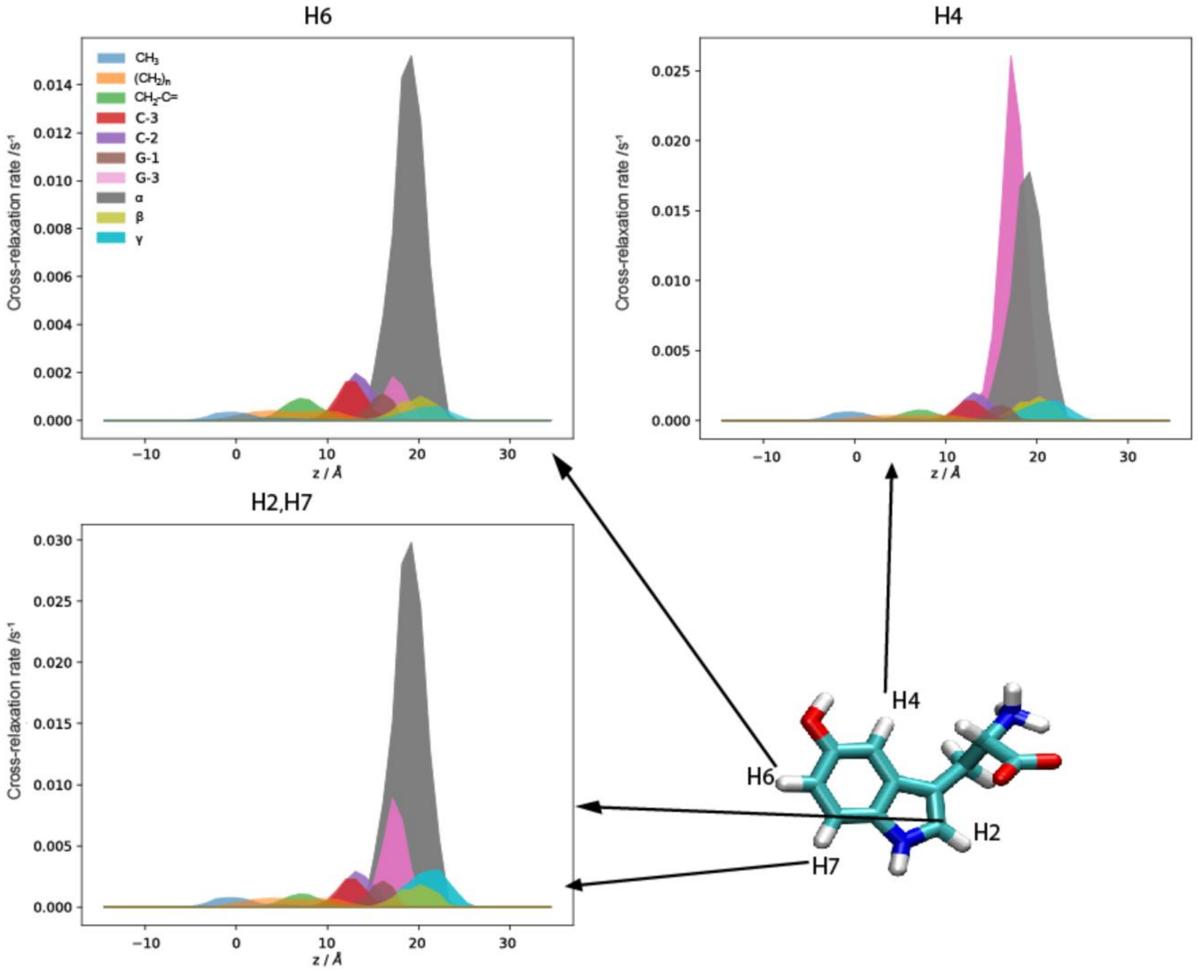

Figure S6. Membrane distributions of 20 mol% 5-HTP in POPC as measured by ^{1}H NMR NOESY NMR under MAS conditions. Shown for all protons measured. For each NOE rate measured, we display the depth distribution of the given functional group on POPC, and scale that distribution by the measured NOE rate constant. Zero on the x-axis corresponds to the mean position of the lipid methyl groups. Certain NOEs were excluded because of intramolecular interactions. The POPC membranes were hydrated to 50 wt% using K_2HPO_4 buffer.

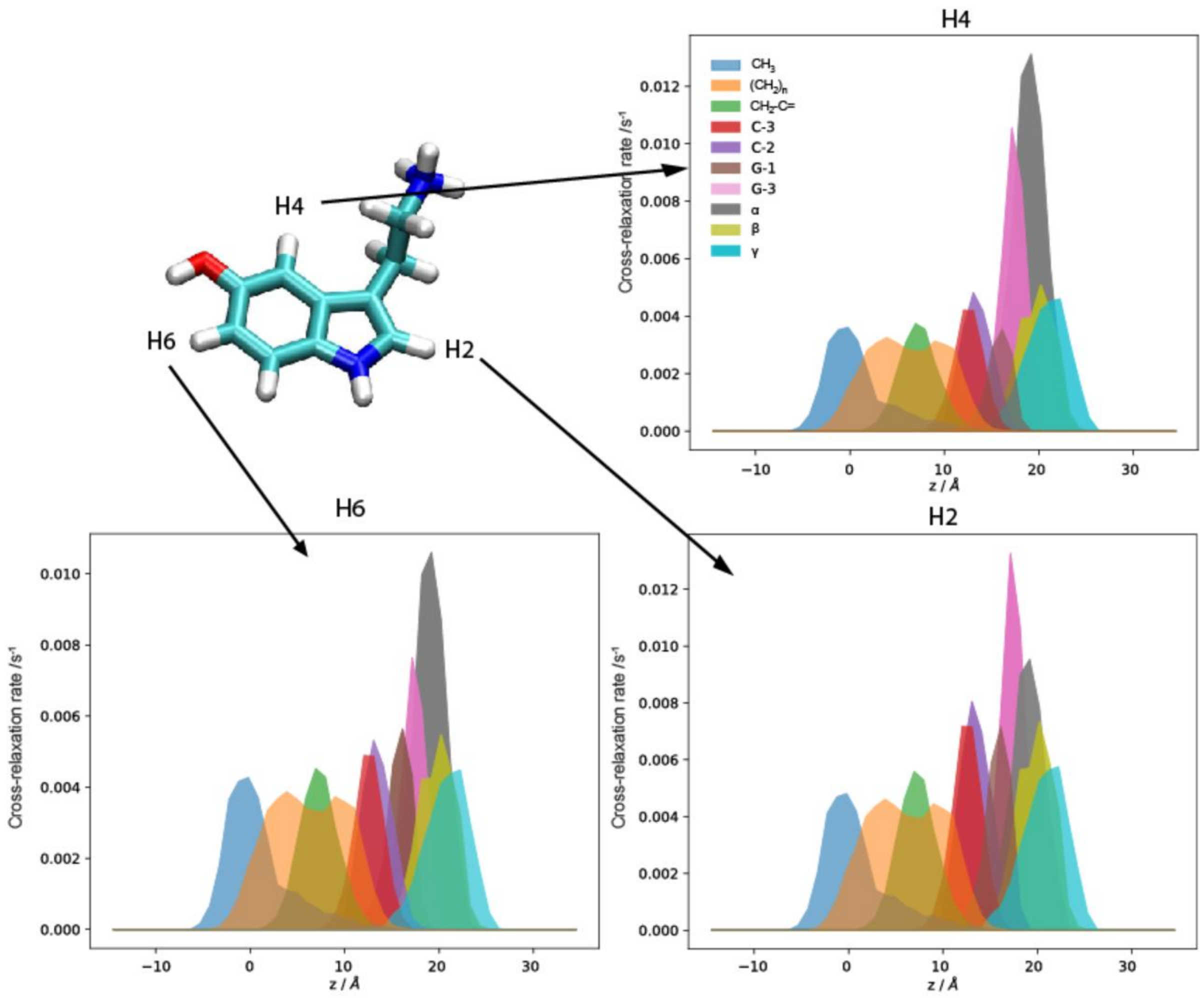

Figure S7. Membrane distributions of 20 mol% serotonin in POPC as measured by ^{1}H NMR NOESY NMR under MAS conditions. Shown for all protons measured. For each NOE rate measured, we display the depth distribution of the given functional group on POPC, and scale that distribution by the measured NOE rate constant. Zero on the x-axis corresponds to the mean position of the lipid methyl groups. Certain NOEs were excluded because of intramolecular interactions. The serotonin data is reproduced from[14]. The POPC membranes were hydrated to 50 wt% using K_2HPO_4 buffer.

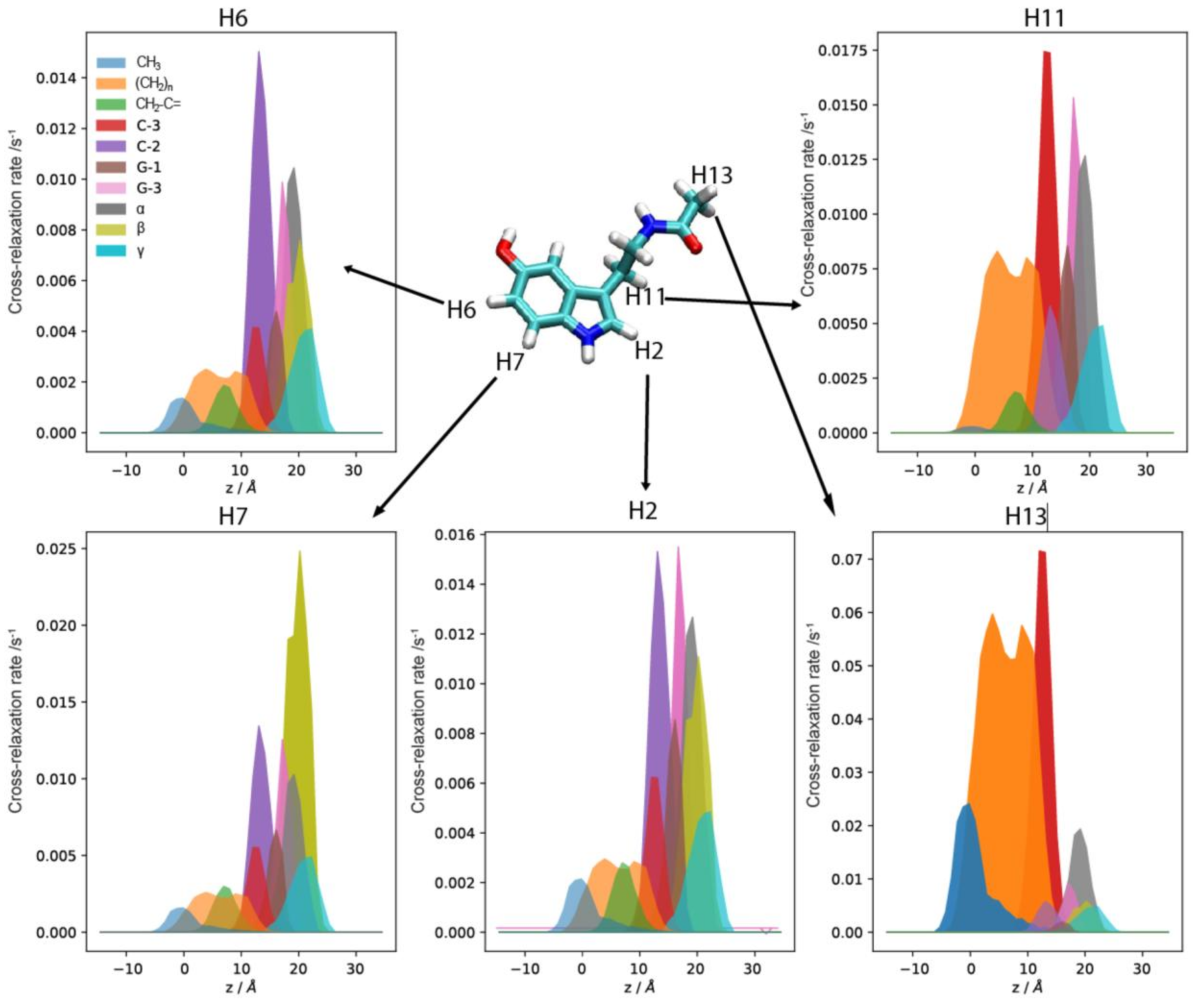

Figure S8. Membrane distributions of 20 mol% NAS in POPC as measured by ^{1}H NMR NOESY NMR under MAS conditions. Shown for all protons measured. For each NOE rate measured, we display the depth distribution of the given functional group on POPC, and scale that distribution by the measured NOE rate constant. Zero on the x-axis corresponds to the mean position of the lipid methyl groups. Certain NOEs were excluded because of intramolecular interactions. The POPC membranes were hydrated to 50 wt% using K_2HPO_4 buffer.

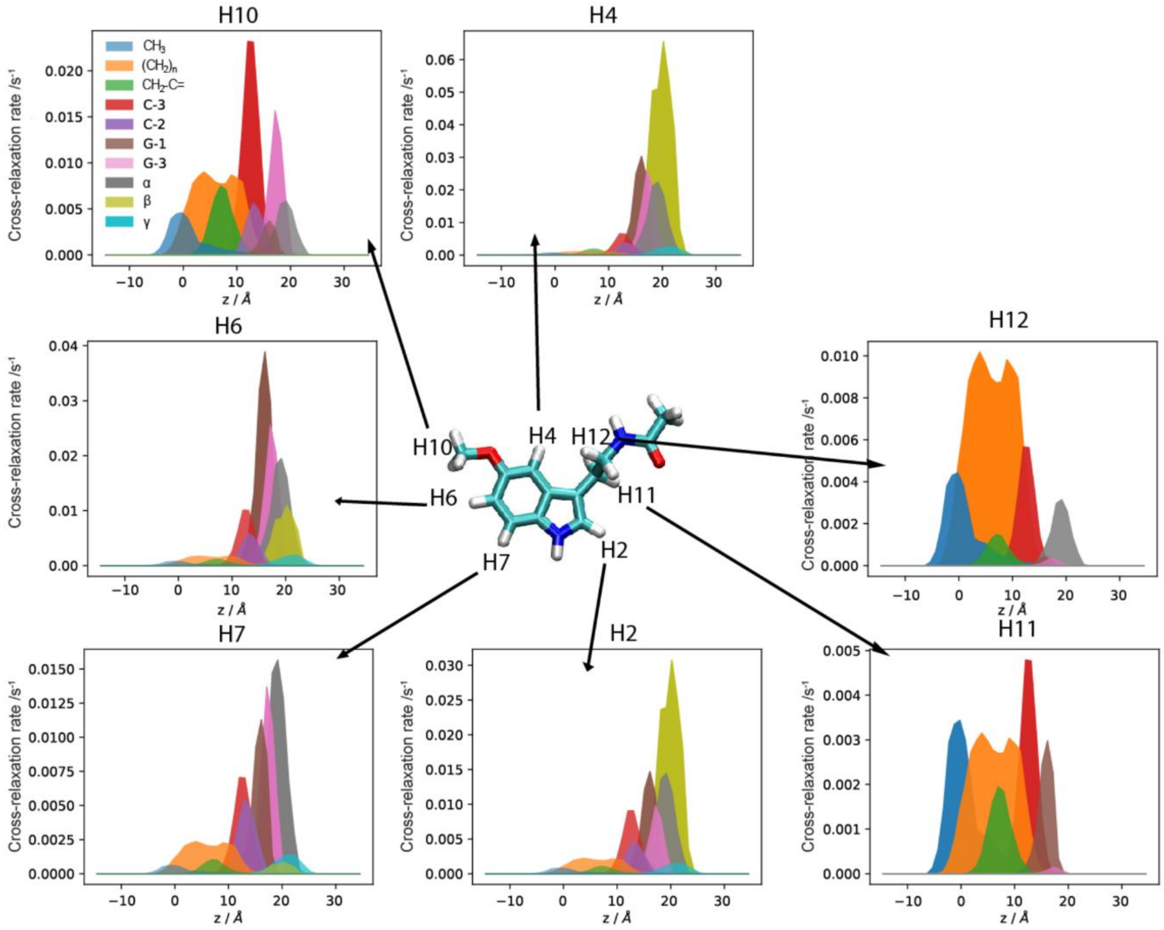

Figure S9. Membrane distributions of 20 mol% melatonin in POPC as measured by ^{1}H NMR NOESY NMR under MAS conditions. Shown for all protons measured. For each NOE rate measured, we display the depth distribution of the given functional group on POPC, and scale that distribution by the measured NOE rate constant. Zero on the x-axis corresponds to the mean position of the lipid methyl groups. Certain NOEs were excluded because of intramolecular interactions. The POPC membranes were hydrated to 50 wt% using K_2HPO_4 buffer.

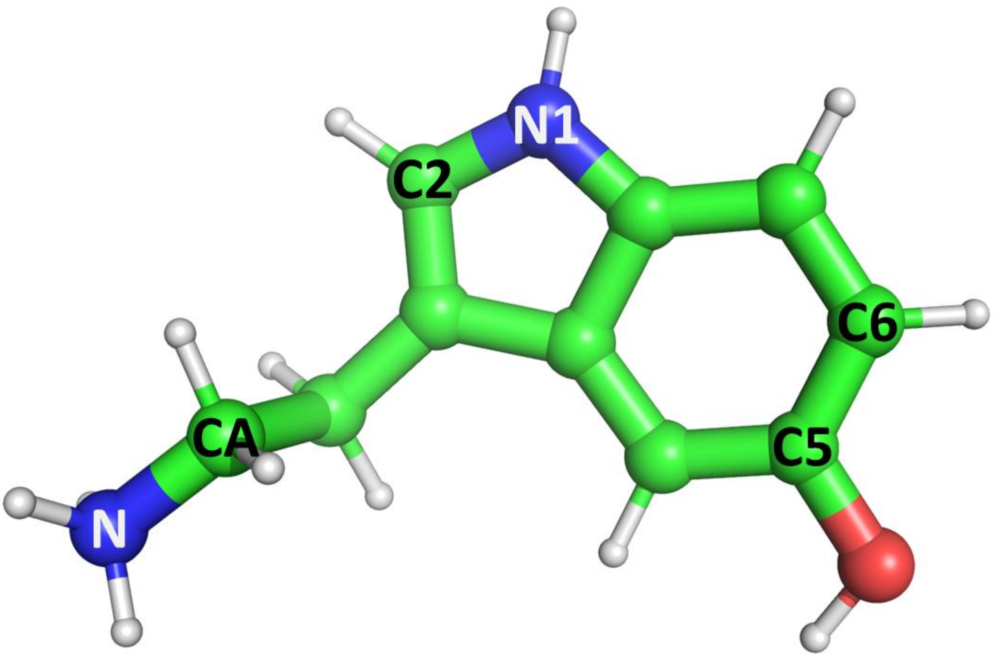

Figure S10. Schematic representation of the serotonin molecule with selected atom names (which were used during H-bond and angle analyses presented on top of them.

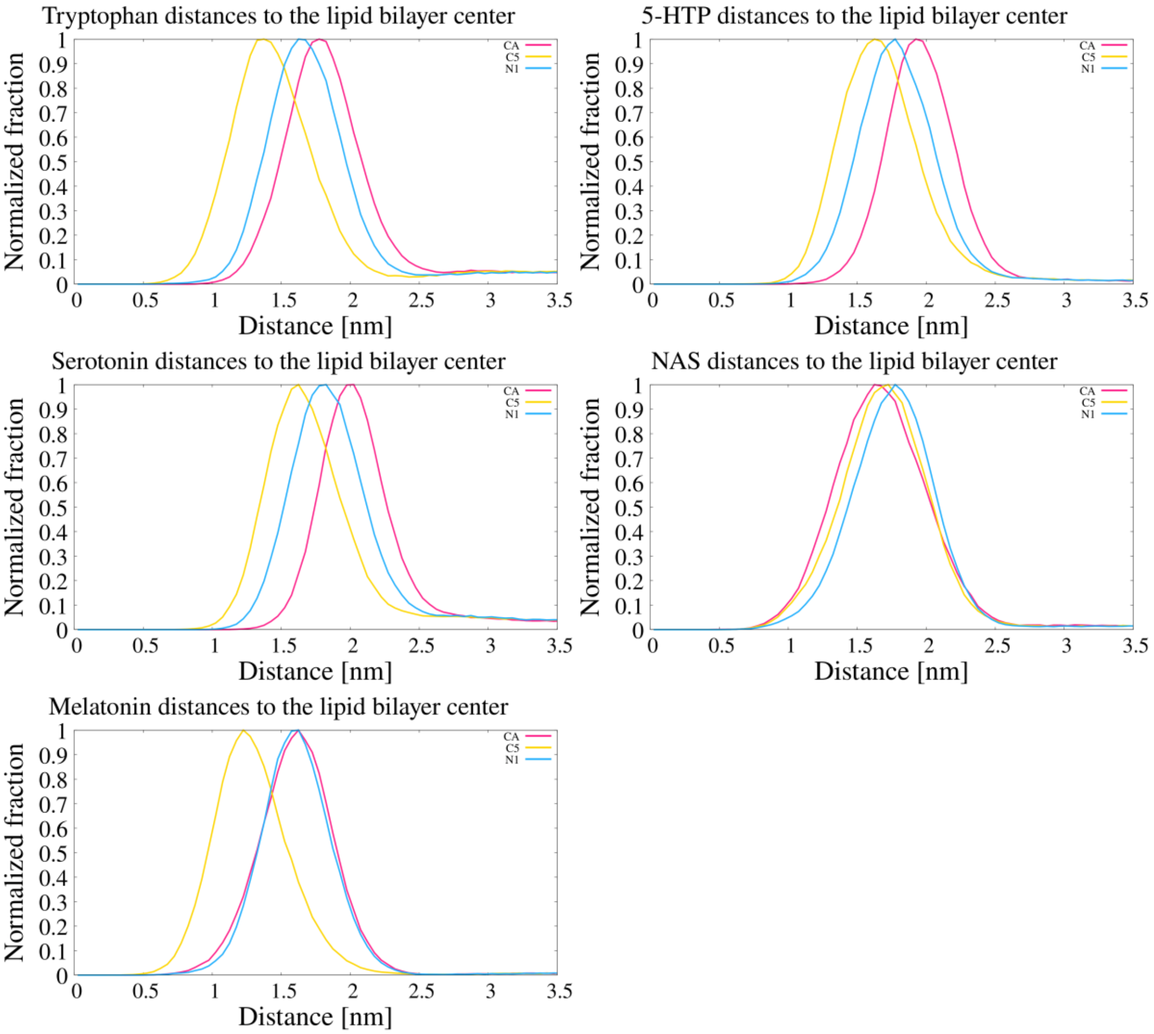

Figure S11. Normalized fraction of neurotransmitters, calculated as the cumulative occurrence of 20 molecules at a given distance from the MD simulations. This distance is defined as the lowest distance between selected heavy atoms (CA red, C yellow, N1 blue, see Figure S10 for explanation) of each of 20 neurotransmitters and the center of the lipid bilayer simulated at 37°C. The normalization was performed by dividing the population of each bin (0.05 nm) by the population of the most populated bin, calculated separately for each molecule. The simulated lipid bilayer was composed of a 3/5/2/5 molar ratio of POPC/POPE/POPS/cholesterol.

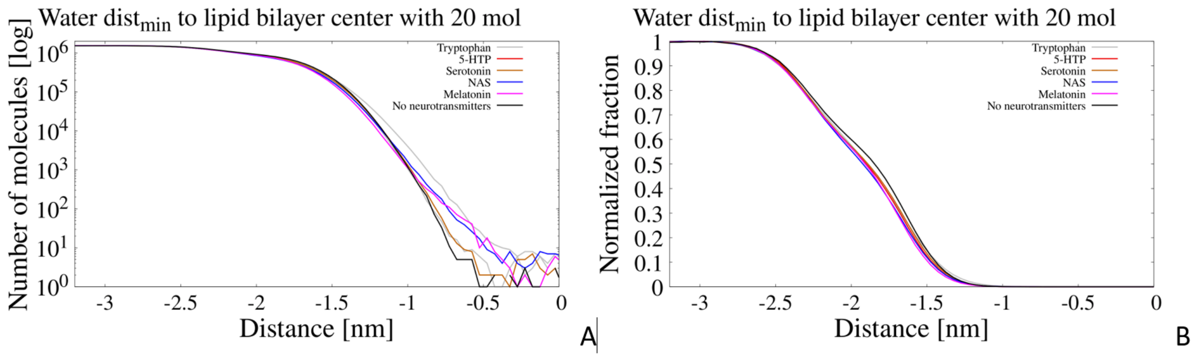

Figure S12. Distribution of water molecules as a function of distance from the lipid bilayer center, shown on a logarithmic scale (panel A) and as normalized values on a linear scale (panel B), calculated from the MD simulations. The simulated lipid bilayer was composed of a 3/5/2/5 molar ratio of POPC/POPE/POPS/cholesterol.

Table S1: Dipole moment amplitudes of the serotonin metabolites calculated in the aqueous continuum and gaseous phase. The decrease in dipole moment from aqueous to gaseous medium is presented in the last column.

Molecule	Charge	Medium	Dipole moment (D)	Dipole moment change (D)
Tryptophan	zwitterionic	Aqueous	5.53	2.60
		Gaseous	2.93	
5-hydroxy tryptophan (5-HTP)	zwitterionic	Aqueous	11.65	8.87
		Gaseous	2.78	
Serotonin	Neutral/charged	Aqueous	5.39	1.62
		Gaseous	3.77	
N-acetyl serotonin (NAS)	Neutral	Aqueous	2.65	1.09
		Gaseous	1.55	
Melatonin	Neutral	Aqueous	4.17	1.31
		Gaseous	2.86	

Table S2: Lifetime components and their relative amplitude found for the serotonin metabolites in the presence and absence of SUVs mimicking the synaptic membranes.

Sample	a1	t1 (ns)	a2	t2 (ns)	a3	t3 (ns)
**Tryptophan**	0.84 ±0.01	2.98 ±0.02	0.16 ±0.01	0.64 ±0.13		
**Tryptophan + SUV**	0.49 ±0.02	2.98	0.41 ±0.02	0.67 ±0.03	0.09 ±0.01	20.03 ±0.93
**5-HTP**	1	3.30 ±0.04				
**5-HTP + SUVs**	0.45 ±0.00	3.30	0.52± 0.00	0.82 ±0.03	0.03 ±0.00	15.35 ±1.85
**Serotonin**	1	3.68 ±0.01				
**Serotonin + SUVs**	0.40 ±0.03	3.68	0.58 ±0.04	0.89 ±0.03	0.01 ±0.01	14.72 ±2.49
**NAS**	1	3.98 ±0.02				
**NAS + SUVs**	0.56 ±0.03	3.98	0.36 ±0.03	0.89 ±0.01	0.08 ±0.01	21.12 ±1.01
**Melatonin**	1	5.09±0.04				
**Melatonin + SUVs**	0.37 ±0.04	5.1	0.62 ±0.04	1.07 ±0.23		

Table S3. Calculated average numbers of hydrogen bonds between N1 and N serotonin metabolites and lipids for all donor-acceptor pairs (total) and N and N1 groups separately, solvent-accessible surface area (SASA, Å²) of the serotonin metabolites, buried SASA (defined as the difference between solution SASA (SASAmax) and SASA from MD simulations, Å²), and total lipid area (XY-area, Å²). Standard deviations are given in brackets.

		**Tryptophan**	**5-HTP**	**Serotonin**	**NAS**	**Melatonin**	**No metabolites**
Hbonds	Total	14.48 (3.53)	28.67 (5.09)	18.18 (4.06)	13.36 (3.41)	7.71 (2.82)	
	Per molecule	0.72	1.43	0.91	0.67	0.39	
	Total_N1 (indole)	3.31 (1.74)	4.93 (1.92)	4.56 (1.89)	3.15 (1.68)	3.24 (1.74)	
	Total_N	1.07 (1.02)	7.20 (2.37)	8.22 (2.77)	2.74 (1.54)	3.10 (1.67)	
SASA	Total	23.53	24.76	8.89	20.22	17.66	
	Per molecule	1.18	1.19	0.44	1.01	0.88	
Buried SASA	Total	42.26	43.99	34.07	48.45	55.88	
	Per molecule	2.11	2.20	1.70	2.42	2.79	
	Percentage	64.1	64.9	79.1	70.6	75.8	
SASAmax	Per molecule	3.29	3.39	2.15	3.43	3.68	
Lipid area	Total	6164.17 (5.61)	6170.75 (6.42)	6167.36 (5.66)	6174.37 (5.85)	6179.26 (5.85)	6133.24 (6.13)
	Percentage increase	0.504	0.612	0.556	0.671	0.750	0
